# Feasibility, validity and reliability of the ASCOT-Proxy and ASCOT-Carer among unpaid carers of people living with dementia in England

**DOI:** 10.1186/s12955-023-02122-0

**Published:** 2023-06-03

**Authors:** Barbora Silarova, Stacey Rand, Ann-Marie Towers, Karen Jones

**Affiliations:** 1 Personal Social Services Research Unit, University of Kent, Cornwallis Central, Canterbury, Kent, CT2 7NF UK; 2 Centre For Health Services Studies, University of Kent, Cornwallis Central, Canterbury, Kent, CT2 7NF UK

**Keywords:** Carers, Dementia, Outcome Assessment, Proxy, Psychometrics, Quality of life, Validation study

## Abstract

**Background:**

People with dementia living at home represent a growing group of social care services users in England. Many are unable to complete questionnaires due to cognitive impairment. The ASCOT-Proxy is an adapted version of an established measure, ASCOT, which was developed as a way of collecting social care-related quality of life (SCRQoL) data from this group of service users, either alone or alongside the ASCOT-Carer, a measure of SCRQoL for unpaid carers. The ASCOT-Proxy includes two perspectives, the proxy-proxy perspective (‘My opinion: What I think’) and proxy-person perspective (‘What I think the person I represent thinks’). We aimed to establish the feasibility, construct validity and reliability of the ASCOT-Proxy and ASCOT-Carer, with unpaid carers of people with dementia living at home unable to self-report. We also aimed to establish structural characteristics of the ASCOT-Proxy.

**Methods:**

Cross-sectional data were collected using self-administered questionnaire (paper or online) among unpaid carers living in England between January 2020 and April 2021. Unpaid carers could take part if they supported someone living with dementia who was unable to self-complete a structured questionnaire. The person living with dementia or their unpaid carer had to use at least one social care service. We used the proportion of missing data to establish feasibility, ordinal exploratory factor analysis to establish structural characteristics, Zumbo’s ordinal alpha for internal reliability, and hypothesis testing for construct validity. We also conducted Rasch analysis.

**Results:**

We analysed data for 313 carers (62.4(± 12.0) years, 75.7% (*N*=237) females). We were able to calculate the ASCOT-Proxy-proxy overall score for 90.7% of our sample, the ASCOT-Proxy-person overall score for 88.8% of our sample and in case of the ASCOT-Carer for 99.7% of our sample. As there was an issue with structural characteristics of the ASCOT-Proxy-proxy we conducted Rasch, reliability and construct validity analysis for the ASCOT-Proxy-person and ASCOT-Carer only.

**Conclusions:**

This was a first study to explore psychometric characteristics of the ASCOT-Proxy and ASCOT-Carer with unpaid carers of people with dementia living at home unable to self-report. There are some aspects of the psychometric characteristics of the ASCOT-Proxy and ASCOT-Carer that warrant further investigation in future.

Trial registration

NA

**Supplementary Information:**

The online version contains supplementary material available at 10.1186/s12955-023-02122-0.

## Background

In 2019, it was estimated that one in every fourteen people aged 65 years and over were living with dementia in the United Kingdom (UK). In the UK, around 61% of older people with dementia live in their own homes irrespective of dementia severity [1]. Notably, at the early stages of dementia, most people prefer to continue to live at home [2]. Hence, it is important to understand how best to support people to stay at home and maintain their independence, links with local community, and wellbeing. As dementia progresses, many people find it increasingly difficult to look after themselves and their homes and may need help with their daily activities [3, 4]. The majority of this help is provided by family members, close friends or neighbours (also referred to as unpaid [5], informal [6] or family carers [7]). However, community-based social care services, including home care and day activities, are also important sources of support [8].

The Adult Social Care Outcomes Toolkit (ASCOT) is a suite of self-report [9–11], interview [12], easy read [13] or mixed methods [14] measures designed to measure social-care related quality of life (SCRQoL) of service users and their carers [15]. The measures are preference-based measures [10, 16] that may be used in economic evaluation of social care services (also known as long-term care) [17, 18], interventions or policy. However, it is difficult to collect information using self-report questionnaires from people who have memory or communication difficulties, including people with moderate to severe dementia [14, 19]. To work around this, an adapted version of the ASCOT self-completion version (SCT4), the ASCOT-Proxy has been developed for completion by someone who knows the person well, such as, a close friend or relative [20–22]. The ASCOT-Proxy uses the same eight domains (items) of SCRQoL identified in the original development work and the final version of the ASCOT-SCT4 [10]. However, the item wording, format and response options were adapted to improve the acceptability of the measure to proxy respondents (care workers or unpaid carers) [20, 21]. Based on the development studies of the ASCOT-Proxy [20–22] and informed by Pickard and Knight’s [23] conceptual framework of proxy-response, the ASCOT-Proxy includes two proxy perspectives: proxy-proxy perspective (‘My opinion: What I think’) and proxy-person perspective (‘What I think the person I represent thinks’) in the response options.

There is also another version of the questionnaire called the ASCOT-Carer, which looks at aspects of life that are important to friends and relatives who look after someone with social care support needs. Previous studies have established that the ASCOT-Carer is a valid and reliable measure of SCRQoL among carers in England [15]. However, the study in England only included a relatively small number of carers of people with dementia, so separate subgroup analysis was not possible [15]. As such, this study will address the evidence gap with regard to the measurement properties of the ASCOT-Carer with this subgroup of carers [24, 25].

The aim of the present study was to establish the feasibility, reliability and construct validity of the ASCOT-Proxy and ASCOT-Carer. In addition, we aimed to compare structural characteristics of the ASCOT-Proxy against the original ASCOT-SCT4 (self-completion form) [10]. The structural characteristics of the ASCOT-Carer using the same data as this study is reported elsewhere [26]).

## Methods

### Participants and setting

Data analysed in this paper were collected as part of the ‘Measuring Outcomes of People with Dementia and their carers’ (MOPED) study. Data were collected using a self-administered questionnaire from unpaid carers in England between 27^th^ January 2020 and 30^th^ April 2021. Participants could choose between a postal or online version (using Qualtrics XM Platform™). The online version asked participants if they wished to leave the question blank, but completing items was not mandatory.

Carers were eligible to take part in the MOPED study if they provided unpaid support to a relative, partner/spouse or friend with dementia, living at home (not in residential or nursing care), who was unable to self-complete a structured questionnaire, even with help. The person living with dementia or their unpaid carer had to use at least one community-based social care service at the time of recruitment to the MOPED study. This was adapted from ‘at the time of recruitment’ to ‘before the start of the COVID-19 pandemic’, since some people temporarily stopped receiving social care support or had reduced or adapted support as a result of the legal restrictions on social interaction due to the pandemic [27]. These services could be funded via the local authority, voluntary (third) sector organisations, or paid for by the person with dementia or their carer. Social care was defined as any type of community-based social care support, including equipment and homes adaptations, information and advice, home care, day activities, or support from carers’ organisations. We included people irrespective of type and frequency of the social care community support received, as in previous research [10, 11]. Carers had to be able to provide informed consent and respond to a self-completion format questionnaire in English.

We recruited participants via Join Dementia Research (JDR), an online panel of volunteers interested in dementia research including people living with dementia, unpaid carers and healthy participants. In addition to JDR, we recruited participants via 25 National Health Service (NHS) research sites.

### Measures

The ASCOT-Proxy [21] is an adapted version of the ASCOT-SCT4 [10], designed to be completed by proxy-report rather than self-report. The eight SCRQoL domains (items) of the ASCOT-SCT4 are also included in the ASCOT-Proxy, with similar wording for the domains (items): food and drink, accommodation cleanliness and comfort, personal cleanliness and comfort, personal safety, social participation and involvement, occupation (doing things a person values and enjoys), control over daily life, and dignity. Each item has four response options that correspond to four different outcome states (ideal state (coded as 3 in the analysis), no unmet needs (coded as 2), some unmet needs (coded as 1), and high-level of unmet needs (coded as 0)). For example for food and drink, the response options are: ‘As clean and comfortable as s/he wants’; ‘Adequately clean and comfortable’; ‘Not quite clean or comfortable enough’, ‘Not at all clean or comfortable’. For occupation, the response options are: ‘Is able to spend his/her time as s/he wants, doing the things s/he values or enjoys’; ‘Is able to do enough of the things s/he values or enjoys with his/her time’; ‘Does some of the things s/he values or enjoys with his/her time, but not enough’; ‘Doesn’t do anything s/he values or enjoys with his/her time’.

The key difference between the ASCOT-Proxy and ASCOT-SCT4 is that the ASCOT-Proxy is designed for completion on behalf of services users by someone who knows the person well, typically a family member or care staff [20, 21], rather than self-report [10]. In the MOPED study, we collected the ASCOT-Proxy data from unpaid carers only, not care staff. The questionnaire is available from the ASCOT website (www.pssru.ac.uk/ascot) [28].

The full details on how we calculated the scores for the ASCOT-Proxy (both perspectives) is provided in the ASCOT-SCT4 Proxy guidance (pages 6-9, [29]). As the ASCOT is a preference-weighted measure of SCRQoL [10, 16] for use in social care research and economic evaluation [17, 18], we converted the raw scores per each item into the preference-weighted values, reflecting their relative importance/value to the general population [10]. We then added together the preference-weighted values and entered them into a formula (overall score = (0.203 x weighted score) – 0.466) to give the overall score [29]. The overall score ranges from -.17 to 1.00. Higher scores indicate better SCRQoL. The formula is based on a Time Trade Off exercise with members of the public [10]. Thus, while a score of 1.00 would mean that the person has reported the ideal state in all domains (items), a score of 0.00 is, in the view of the general population, valued the same as being dead. Scores, and the states that they represent, between -0.01 and -0.17 are seen as being ‘worse than death’. A negative score will be calculated, for example, if a person has high unmet needs in all domains (items) (e.g. no control over daily life, not at all clean or comfortable, socially isolated, not enough food and drink etc.). During the preference study, people said death would be preferable to living in this state.

The ASCOT-Carer-SCT4 is a measure of SCRQoL for unpaid carers, aged 18 years or over [15]. It includes seven domains (items), one each for the following SCRQoL attributes: feeling encouraged and supported, space and time to be yourself, self-care, personal safety, social participation and involvement, occupation (doing things a person values and enjoys) and control over daily life. Each domain (item) has four response options, like the other ASCOT measures, reflecting four different outcome states (ideal state (coded as 3 in analysis), no unmet needs (coded as 2), some level of unmet needs (coded as 1), and high-level of unmet needs (coded as 0)). For example, the response options for occupation are: ‘I’m able to spend my time as I want, doing things I value or enjoy’; ‘I’m able to do enough of the things I value or enjoy with my time’; ‘I do some of the things I value or enjoy with my time, but not enough’; ‘I don’t do anything I value or enjoy with my time’. For self-care the response options are: ‘I look after myself as well as I want’; ‘I look after myself well enough’; ‘Sometimes I can’t look after myself well enough’; ‘I feel I am neglecting myself’.

The questionnaire is also available from the ASCOT website (www.pssru.ac.uk/ascot) [30].

The full details on how we calculated the scores for the ASCOT-Carer is provided in the ASCOT-Carer-SCT4 guidance (pages 5-7 [31]). The ASCOT-Carer is a preference-weighted measure of carers’ SCRQoL. We converted the raw scores for each item into the preference-weighted value, based on best-worst scaling [16]. These reflect their relative importance/value to the general population. The overall SCRQoL score for carers is calculated by summing the preference-weighted values for each domain. The overall score ranges between 0 (high-level of unmet needs) and 1 (ideal state).

The full detail of measures used for establishing construct validity is reported in Additional File 1.

### Statistical analysis

Analyses were conducted in Stata 16 [32] and in WINSTEPS software (version 3.92.1.). The full detail of the descriptive analysis, feasibility, structural characteristics, Rasch analysis, reliability/internal consistency, construct validity, and sample size calculation is reported in Additional File 2.

## Results

### Descriptive analysis

**Fig. 1:**
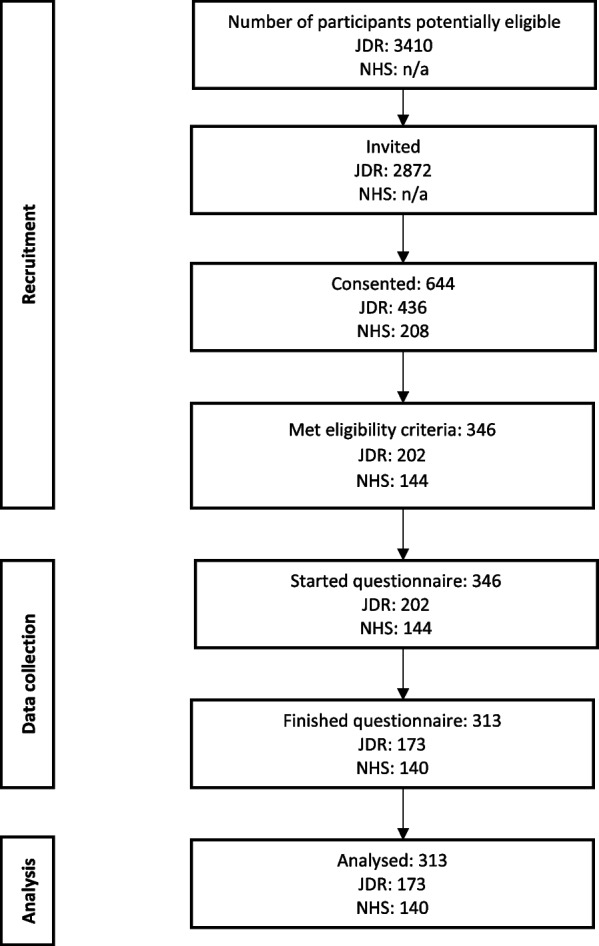
Flow of the participants through the study. Abbreviations: JDR (Join Dementia Research); NHS (the National Health Service). Notes: n/a (information was not available)

The flow of participants through the MOPED study is shown in Fig 1. Characteristics of participants (*n*=313 carers) are shown in Table 1.

**Table 1:** Sample Characteristics

**Variable**	**Total**	**Mean (SD)/Median (IQR)/% (N)**
**Sociodemographic characteristics**		
Age: Carer	313	62.44 (12.04)
Age: Person with dementia	312	81.47 (9.37)
Gender: Carer	313	
Female		75.72% (237)
Male		24.28% (76)
Ethnicity: Carer	313	
White/White British		94.57% (296)
Multiple or mixed ethnic groups		0.96% (3)
Asian/Asian British		1.92% (6)
Black/African/Caribbean/Black British		1.60% (5)
Other Ethnic group		0.96% (3)
Region	303	
Greater London		12.78% (40)
South East		31.95% (100)
South West		11.50% (36)
East of England		6.39% (20)
East Midlands		4.79% (15)
West Midlands		7.03% (22)
Yorkshire and Humber		10.22% (32)
North West		9.58% (30)
North East		2.56% (8)
I/ADLS: proxy-proxy (number of ADLs with difficulty or unable to complete alone)	310	Median: 5 (IQR: 3; 8)
**Caregiving situation**		
Lives in the same household	313	
Yes		57.83% (181)
No		42.17% (132)
Relationship to a person living with dementia	313	
Husband/wife/partner		41.53% (130)
Parent (mother, father)		48.88% (153)
Sibling		1.28% (4)
Other		8.31% (26)
Hours of care per week	310	
50 or more		46.96% (147)
49 and less		52.08% (163)
Help with personal care	311	
Yes		73.80% (231)
No		25.56% (80)
Help with giving medicines	311	
Yes		80.51% (252)
No		18.85% (59)
**Impact of caring on health**	311	
Yes		94.57% (296)
No		4.79% (15)
**Home design suitability: proxy-proxy perspective**	313	
Meets their needs very well		32.27% (101)
Meets most of their needs		46.65% (146)
Meets some of their needs		17.89% (56)
Totally inappropriate		3.19% (10)
**Satisfaction with social care services: Carer (7-point scale)**	309	Median: 5 (IQR: 4; 6)
**Well-Being & Health**		
EQ-5D-5L: Carer	313	Median: 0.77 (IQR: 0.71; 0.88)
EQ-5D-5L: Proxy-proxy	311	0.26 (0.28)
EQ-5D-5L: Proxy-person	309	Median: 0.53 (IQR: 0.09; 0.74)
C-DEMQOL: Carer	304	86.22 (18.86)
DEMQOL: Proxy-proxy	291	Median: 87 (IQR: 76; 99)
Overall quality of life: Carer	312	Median: 4 (IQR: 3; 4)
Overall quality of life: Proxy-proxy	310	Median: 3 (IQR: 2; 4)
Overall quality of life: Proxy-person	310	Median: 3 (IQR: 2; 4)
Carer Experience Scale (CES): Carer	309	Median: 64.38 (IQR: 49.63; 77.55)
**Mode of administration**	313	
Online		20.13% (63)
Paper		79.87% (250)

Only non-missing data are presented and therefore % do not add up to 100%.

*Abbreviations*: *IQR* Interquartile range, *SD* Standard deviation

The distribution of responses as well as overall scores for the ASCOT-Proxy (both perspectives) and ASCOT-Carer are shown in Tables 2 and 3.

**Table 2:** Distribution of responses for the ASCOT Proxy: both perspectives (*n*=313)

	**Outcome states/response options % (N)**				
**Social care related-quality of life attribute**	**Ideal state**	**No unmet needs**	**Some unmet needs**	**High-level of unmet needs**	**Missing**
**Food and drink**					
Proxy-Proxy	62.62% (196)	20.77% (65)	9.27% (29)	6.39% (20)	0.96% (3)
Proxy-Person	67.73% (212)	20.13% (63)	9.90% (31)	0.32% (1)	1.92% (6)
**Accommodation cleanliness and comfort**					
Proxy-Proxy	61.34% (192)	27.80% (87)	9.58% (30)	1.28% (4)	n/a
Proxy-Person	74.76% (234)	19.49% (61)	4.15% (13)	0.32% (1)	0.32% (4)
**Personal cleanliness and comfort**					
Proxy-Proxy	42.17% (132)	43.45% (136)	12.78% (40)	1.60% (5)	n/a
Proxy-Person	70.93% (222)	23.96% (75)	4.15% (13)	0.32% (1)	0.64% (2)
**Personal safety**					
Proxy-Proxy	58.47% (183)	29.07% (91)	28% (8.95)	2.88% (9)	0.64% (2)
Proxy-Person	58.15% (182)	24.28% (76)	12.14% (38)	3.83% (12)	1.60% (5)
**Social participation and involvement**					
Proxy-Proxy	14.38% (45)	22.36% (70)	42.49% (133)	20.45% (64)	0.32% (1)
Proxy-Person	21.09% (66)	28.43% (89)	28.12% (88)	21.41% (67)	0.96% (3)
**Occupation**					
Proxy-Proxy	7.67% (24)	17.25% (54)	44.41% (139)	30.35% (95)	0.32% (1)
Proxy-Person	14.38% (45)	24.60% (77)	34.19% (107)	25.88% (81)	0.96% (3)
**Control over daily life**					
Proxy-Proxy	13.10% (41)	30.67% (96)	25.56% (80)	29.71% (93)	0.96% (3)
Proxy-Person	20.77% (65)	24.92% (78)	23.32% (73)	29.39% (92)	1.60% (5)
**Dignity**					
Proxy-Proxy	29.07% (91)	43.13% (135)	17.25% (54)	3.83% (12)	6.71% (21)
Proxy-Person	21.41% (67)	36.42% (114)	27.48% (86)	7.67% (24)	7.03% (22)
**ASCOT Proxy: overall scores (range: -0.17 – 1)**	**Total**	**Median**	**IQR**	**% (N) min value**	**% (N) max value**
Proxy-Proxy	284	0.63	0.21; 0.76	0% (0)	1.3% (4)
Proxy-Person	278	0.68	0.48; 0.81	0% (0)	1.3% (4)

*Abbreviations*: *IQR *Interquartile range, *SD *Standard deviation, *n/a* No missing data

**Table 3:** Distribution of responses for the ASCOT Carer (*n*=313)

	**Outcome states/response options % (N)**				
**Social care related-quality of life attribute**	**Ideal state**	**No unmet needs**	**Some unmet needs**	**High-level of unmet needs**	**Missing**
Feeling encouraged and supported	13.42% (42)	36.42% (114)	38.98% (122)	11.18% (35)	n/a
Space and time to be yourself	7.03% (22)	30.35% (95)	53.67% (168)	8.95% (28)	n/a
Self-care	21.09% (66)	46.96% (147)	18.53% (58)	13.42% (42)	n/a
Personal safety	75.08% (235)	21.41% (67)	2.56% (8)	0.64% (2)	0.32% (1)
Social participation and involvement	13.42% (42)	27.80% (87)	44.73% (140)	14.06 (44)	n/a
Occupation	5.11% (16)	23.32% (73)	66.13% (207)	5.43% (17)	n/a
Control over daily life	10.22% (32)	38.02% (119)	45.37% (142)	6.39% (20)	n/a
	**Total**	**Median**	**IQR**	**% (N) min value**	**% (N) max value**
**ASCOT Carer: overall score (range: 0– 1)**	312	0.66	0.49; 0.83	0% (0)	1.6% (5)

*Abbreviations*: *IQR* Interquartile range, *SD* Standard deviation, *n/a* No missing data

### Feasibility

Overall, the percentage of missing values was low for the ASCOT-Proxy (both perspectives) ranging from 0% to 1.92% (Table 2). The dignity item had a higher percentage of missing data (Proxy-proxy: ‘My opinion: What I think’: 6.71%; proxy-person: ‘What I think the person I represent thinks’: 7.03% of missing data). The overall ASCOT-Proxy-proxy score could be calculated for 90.7% of our sample while the overall ASCOT-Proxy-person score could be calculated for 88.8% of our sample. Immediate form of one-sample test of proportion confirmed that there was no significant difference (*p*= 0.258) in the proportion of missing data between the ASCOT-Proxy-proxy (29/313) and ASCOT-Proxy-person (35/313) overall scores. This indicates that both ASCOT-Proxy perspectives have similar feasibility for unpaid carers of people living with dementia.

We explored whether the feasibility of the ASCOT-Proxy perspectives differ based on mode of administration (paper version versus online) using the immediate form of two-sample test of proportions to compare the proportion of missing data for the ASCOT-Proxy scores by mode of administration. For the ASCOT-Proxy-proxy perspective, the proportion of missing data was significantly (*p*<0.001) higher for participants who used paper questionnaires (missing: 13/63) when compared to those using online version (missing: 16/250). For the ASCOT-Proxy-person perspective, the proportion of missing data was also significantly (*p*<0.001) higher for participants who used paper questionnaires (missing: 17/63) when compared to those using online version (missing: 18/250).

Regarding the ASCOT-Carer, all but one item (personal safety; 0.32% of missing data) had no missing data indicating a good feasibility (Table 3). We were able to calculate the ASCOT-Carer overall score for 99.7% of our sample. As only one observation was missing for the ASCOT-Carer overall score, we did not explore the role of administration mode in acceptability of the ASCOT-Carer.

### Factor structure of ASCOT-Proxy perspectives

**Fig. 2:**
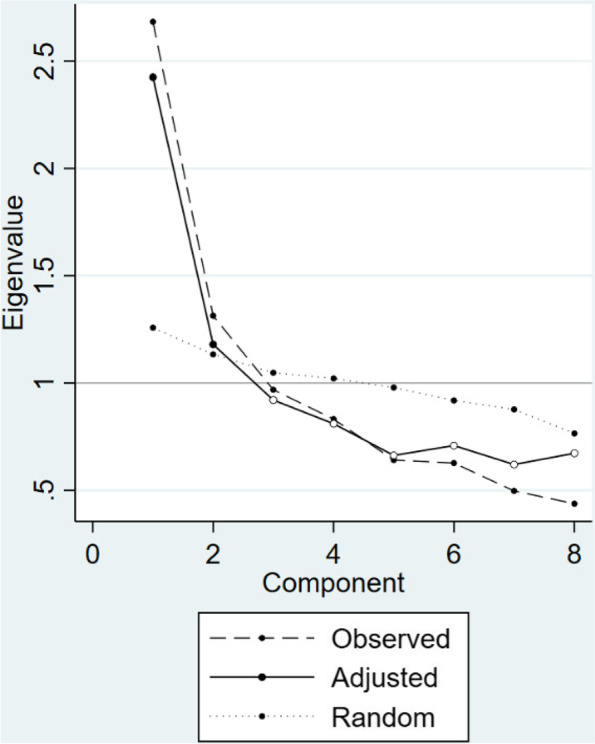
Parallel analysis of the ASCOT-Proxy-proxy. Note: Plot of actual principal component eigenvalues versus randomly generated 95th percentile eigenvalues

As the ASCOT-Proxy has Likert-type items which yield ordinal data, we undertook several steps when examining structural characteristics of the ASCOT-Proxy (both perspectives) as recommended by Gugiu et al. [33]. First, based on the results from Horn’s parallel analysis (principal component analysis as a factor estimation type, 5000 iterations, using 95th percentile for randomly generated eigenvalues, similarly as in a previous study [34]), we retained two factors for the ASCOT-Proxy-proxy (Fig. 2). To inform our decision regarding the number of factors to retain, we compared the observed principal component eigenvalues with the 95th percentile eigenvalues (random) from the simulated datasets. We retained those factors where observed eigenvalues exceeded the eigenvalues generated by random.

Then, we performed maximum likelihood exploratory factor analysis on the polychoric correlation matrix (ordinal exploratory factor analysis). In case of the ASCOT-Proxy-proxy we specified to retain two factors and applied oblique rotation (promax) to allow for the factors to be correlated. Next, we verified that the suggested solution from the parallel analysis, supported the stability and interpretability of the factors structure. We checked whether any items had low factor loadings (<.4) on all the factors, or salient loadings (>.5) on two factors [33]. Based on this (see Table 4) we retained two factor solution in case of the ASCOT-Proxy-proxy. These two factors can represent (1) basic domains (items) that relate to basic care needs/support to sustain life and health (food and drink, accommodation cleanliness and comfort, personal cleanliness and comfort, personal safety) and 2) higher order domains (items) that relate to aspects beyond basic care needs and/or relate to a person’s sense of self and identity (social participation and involvement, occupation, control over daily life) [10]. While we consider our findings from parallel analysis robust (the real eigenvalue line for two factors is above the 95th percentile eigenvalue for the simulated data), it is the first time when the results indicate that one of the ASCOT tools has different structure than other ASCOT tools. For example, the original ASCOT-SCT4 [10] has a weak unidimensional one-factor structure which was also confirmed for other adapted version, e.g. easy-read ASCOT [13] and the translation of ASCOT into Finnish [35].

**Table 4:** Rotated factor loadings and unique variances for the ASCOT-Proxy-proxy

**Item**	**Factor 1: Basic domains**	**Factor 2: Higher order domains**	**Uniqueness**
Food and drink	**0.6006**	0.0006	0.6390
Accommodation cleanliness and comfort	**0.8372**	-0.0442	0.3298
Personal cleanliness and comfort	**0.8472**	-0.1115	0.3531
Personal safety	**0.5977**	0.0992	0.5806
Social participation and involvement	0.2664	**0.4215**	0.6524
Control over daily life	-0.0473	**0.4773**	0.7898
Occupation	-0.1014	**0.9939**	0.0906
Dignity	0.2020	0.0399	0.9505

Factor 1 represents (1) basic domains (items) that relate to basic care needs/support to sustain life and health (food and drink, accommodation cleanliness and comfort, personal cleanliness and comfort, personal safety). Factor 2 represents higher order domains that relate to aspects of quality of life beyond basic care needs and/or relate to a person’s sense of self and identity (social participation and involvement, occupation, control over daily life).

The correlation between the promax rotated factors was: 0.4407. This suggests that promax rotation is an acceptable solution (the correlation was higher than 0.30).

**Fig. 3:**
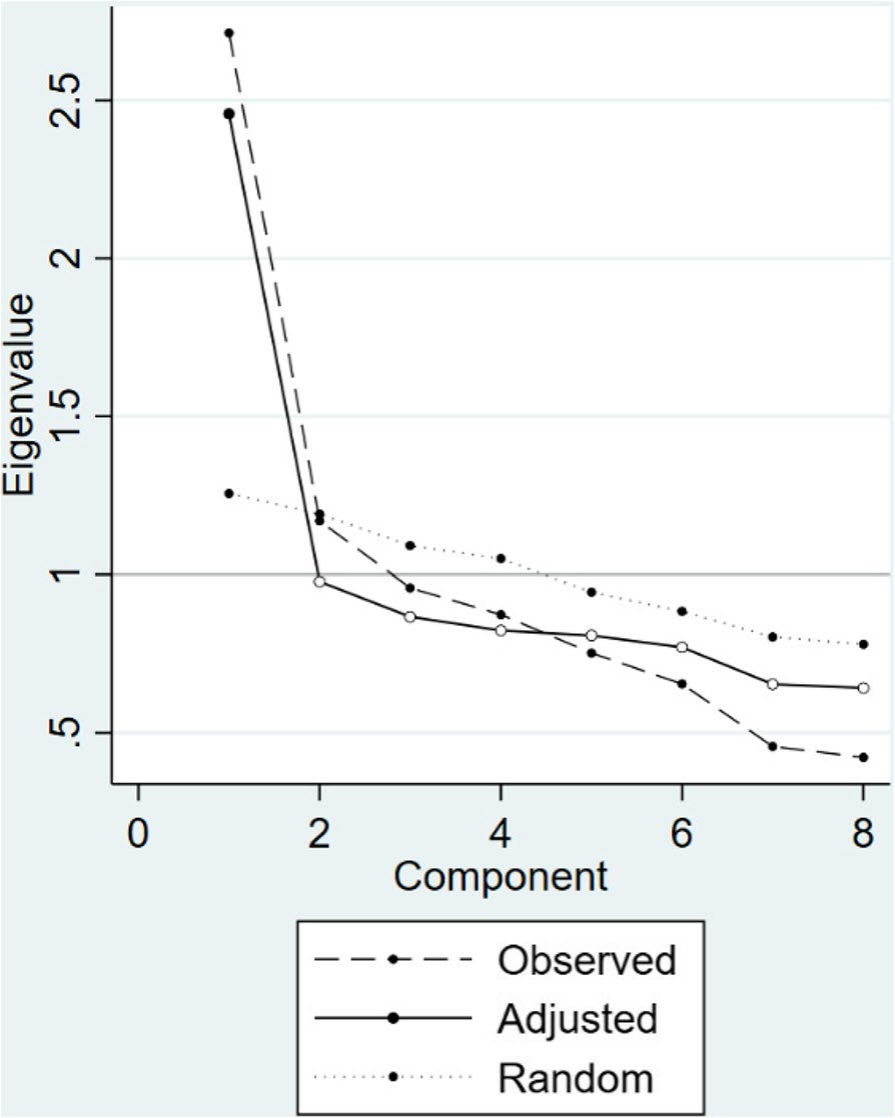
Parallel analysis of the ASCOT-Proxy-person. Note: Plot of actual principal component eigenvalues versus randomly generated 95th percentile eigenvalues

In case of the ASCOT-Proxy-person, the suggested solution from the parallel analysis was to retain one factor (Fig. 3). However, given the close proximity between the random eigenvalue line and the observed eigenvalues for two factors, the result from the parallel analysis suggests that in a different sample, one may potentially find a two factor solution. Therefore, we ran maximum likelihood exploratory factor analysis on the polychoric correlation matrix (ordinal exploratory factor analysis) and specified to retain one factor and then two factors (oblique rotation - promax). Next, we checked whether any items had low factor loadings (<.4) on all the factors, or salient loadings (>.5) on two factors [33]. As one factor solution better supported the stability and interpretability of the factor structure, we conclude that the ASCOT-Proxy-person has a weak unidimensional (one-factor) structure (Table 5).

**Table 5:** Factor loadings and unique variances for the ASCOT-Proxy-person: comparison of one factor solution vs two-factor solution

**ASCOT-Proxy-person (original)**	**One factor solution**		**Two-factor solution**		
**Item**	**Factor 1**	**Uniqueness**	**Factor 1**	**Factor 2**	**Uniqueness**
Food and drink	**0.4634**	0.7853	0.3870	0.1032	0.7929
Accommodation cleanliness and comfort	**0.6487**	0.5793	**0.8219**	-0.0724	0.3889
Personal cleanliness and comfort	**0.7286**	0.4692	**0.8547**	0.0085	0.2609
Personal safety	**0.4929**	0.7571	0.2920	0.2420	0.7735
Social participation and involvement	**0.4364**	0.8096	-0.1222	**0.6166**	0.6931
Control over daily life	**0.7076**	0.4993	0.1971	**0.5872**	0.4809
Occupation	**0.7038**	0.5046	-0.0428	**0.9150**	0.2067
Dignity	0.2507	0.9371	0.0367	0.2261	0.9378
**ASCOT-Proxy-person (recoded)**^**a**^	**One factor solution**		**Two-factor solution**		
**Item**	**Factor 1**	**Uniqueness**	**Factor 1**	**Factor 2**	**Uniqueness**
Food and drink: recoded	**0.4160**	0.8269	0.1308	0.3372	0.8162
Accommodation cleanliness and comfort	**0.6534**	0.5731	-0.1252	**0.9521**	0.2209
Personal cleanliness and comfort	**0.7199**	0.4817	0.1257	**0.7005**	0.3877
Personal safety	**0.4898**	0.7601	0.2551	0.2855	0.7659
Social participation and involvement	**0.4477**	0.7996	**0.6373**	-0.1359	0.6794
Control over daily life: recoded	**0.7295**	0.4677	**0.6584**	0.1208	0.4563
Occupation	**0.7054**	0.5024	**0.8855**	-0.0629	0.2789
Dignity	0.2788	0.9222	0.2311	0.0720	0.9214

In case of two-factor solution, we report rotated factor loadings (oblique rotation-promax). The correlation between the promax rotated factors was: 0.5851 for the ASCOT-Proxy-person (original) and 0.6008 for the ASCOT-Proxy-person (recoded). This suggests that promax rotation is an acceptable solution (the correlation was higher than 0.30).

^a^The lowest two (high-level of unmet needs, some needs) and highest two categories (no needs, ideal state) were combined for food and drink and for control over daily life.

**Fig. 4:**
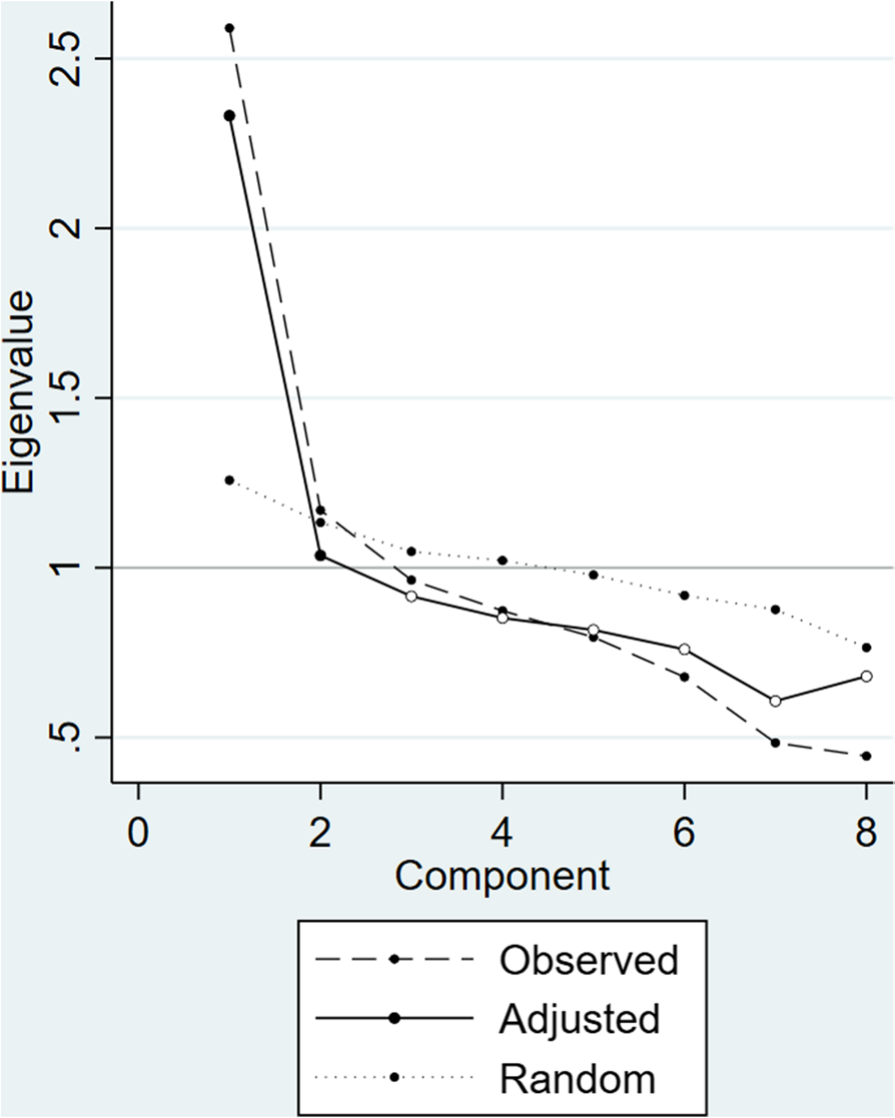
Parallel analysis of the ASCOT-Proxy-person with recoded values for food and drink and control over daily life. Note: Plot of actual principal component eigenvalues versus randomly generated 95th percentile eigenvalues

Rasch analysis (the category probability curves; see below) suggested that the lowest two (high-level of unmet needs, some needs) and highest two categories (no needs, ideal state) should be combined for food and drink and for control over daily life in case of the ASCOT-Proxy-person. As a result of the Rasch findings, we reran the parallel analysis and exploratory factor analysis using the recoded data. The suggested solution from the parallel analysis was to retain two factors (Fig. 4). Therefore, we ran maximum likelihood exploratory factor analysis on the polychoric correlation matrix (ordinal exploratory factor analysis) and specified to retain two factors (oblique rotation - promax). Next, we checked whether any items had low factor loadings (<.4) on all the factors, or salient loadings (>.5) on two factors [33]. Based on the results (Table 5) two factor solution seemed not to be appropriate. Therefore, we reran the exploratory factor analysis and specified to retain one factor and explored the factor loadings of each domain (item). As one factor solution better supported the stability and interpretability of the factor structure, we conclude that the ASCOT-Proxy-person has a weak unidimensional (one-factor) structure (Table 5).

In addition, it is important to highlight that the dignity domain (item) had low factor loadings (<.4) in the ASCOT-Proxy (both perspectives) (Tables 4 and 5). This may indicate that the dignity domain (item) should be dropped from further analysis. However, as highlighted by Gugiu et al. [33], this may be not always the case, e.g. researchers may consider to keep the item if dropping it compromises the content validity of the instrument. The strongest evidence to support the decision whether dropping the item would compromise the content validity comes from qualitative concept elicitations studies. In the ASCOT-SCT4 [10] and therefore ASCOT-Proxy, dignity is defined as ‘the negative and positive psychological impact of support and care on the service user’s personal sense of significance’. It has been identified as an important aspect of SCRQoL based on reviewing previous literature about measures of SCRQoL, as well as a series of qualitative studies with service users [10]. As the ASCOT-Proxy is an adapted version of the ASCOT-SCT4, the dignity domain (item) was considered for inclusion in the development studies of the ASCOT-Proxy [20, 21]. In the early development study of the ASCOT-Proxy, the participants (*n*=35 unpaid carers, *n*=8 care workers) described having greater difficulty answering those ASCOT questions by proxy that related to abstract concepts (e.g. dignity, occupation, control over daily life) than concrete concepts (e.g. food and drink) [20]. This issue was then further explored with 25 unpaid carers and care workers in three iterative rounds of cognitive interviews [21]. Overall, 22/25 participants correctly interpreted the dignity item and 18/25 found the item (in the draft version presented) to be acceptable. The following concerns over acceptability were raised: care staff (as proxies) may not respond accurately, as it may reflect badly on them (*n*=1), it was difficult for the respondent to imagine the person’s perspective on dignity due to condition-specific considerations (e.g., perceived lack of self-awareness) (*n*=3), the person did not currently receive paid help (i.e. it was not applicable) (*n*=2) or another unspecified reason (*n*=1). These may be mitigated by focussing on unpaid carers as proxies and also limiting the sample to those receiving care, as in this study. Given the fact that dignity is an important aspect of SCRQoL as established in the ASCOT-SCT4 [10] and was found acceptable in the development study for the ASCOT-Proxy [21], in this study, we retained it in further analysis. However, it is important that researchers report structural characteristics of the ASCOT-Proxy in the future studies. If the present finding is replicated, it may be necessary to revisit whether dignity should be included as an important aspect of SCRQoL of social care users when the answers are provided by proxy (e.g. through qualitative concept studies with unpaid carers as proxy respondents).

As the results from factor structure analysis inform Rasch analysis, reliability and construct validity analysis, these were only conducted with the ASCOT-Proxy-person in this study. We decided not to proceed with these analysis for the ASCOT-Proxy-proxy as whether and how the ASCOT-Proxy-proxy tool should be used when having two factor solution have to be considered carefully.

The one factor structure of the ASCOT-Carer using the same data as this study has been established elsewhere and is not included in this paper [26].

### Rasch analysis: Overall model fit

For the full description of Rasch analysis please see Additional File 2. We assessed model fit using the information-weighted mean square (INFIT MNSQ), outlier-sensitive mean square (OUTFIT MNSQ) statistics and point-measure correlation. Similarly as in another study [36], we considered values of INFIT and OUTFIT MNSQ statistics in the range of .5 to 1.5 as satisfactory [37]. The INFIT MNSQ and OUTFIT MNSQ statistics for the ASCOT-Proxy-person and ASCOT-Carer are shown in Table 6 and Table 7. All items were in the acceptable INFIT MNSQ and OUTFIT MNSQ range of .5 to 1.5 and consisted of positive item-Rasch measure correlations, except for the ASCOT-Carer personal safety (OUTFIT MNSQ=2.27). This indicates there may be an issue with the item, indicating that the item should be dropped. As this is the first study to use Rasch analysis when exploring psychometric properties of the ASCOT-Carer in unpaid carers, it is important that future studies using the ASCOT-Carer explore this further in their analysis.

**Table 6:** Item statistics including difficulty (in logits), infit and outfit mean square and point-measure correlations for the ASCOT-Proxy-person

	**ASCOT-Proxy-person**					**ASCOT-Proxy-person – collapsed categories**^**b**^				
**Scale domain (item)**	**Item difficulty**	**SE**	**INFIT MNSQ**^**a**^	**OUTFIT MNSQ**^**a**^	**Point-measure correlation**	**Item difficulty**	**SE**	**INFIT MNSQ**^**a**^	**OUTFIT MNSQ**^**a**^	**Point-measure correlation**
Food and drink	-1.38	0.10	1.06	1.28	0.42	-1.38	0.20	0.98	1.06	0.27
Accommodation cleanliness and comfort	-1.53	0.12	0.94	0.95	0.43	-1.61	0.12	0.95	0.91	0.45
Personal cleanliness and comfort	-1.48	0.11	0.88	0.81	0.49	-1.56	0.11	0.92	0.94	0.48
Personal safety	-0.33	0.08	1.12	1.12	0.48	-0.36	0.09	1.09	1.09	0.51
Social participation and involvement	1.18	0.07	1.12	1.25	0.59	1.23	0.07	1.10	1.23	0.63
Control over daily life	1.37	0.07	0.81	0.78	0.72	1.41	0.13	0.82	0.75	0.59
Occupation	1.52	0.07	0.71	0.72	0.75	1.60	0.08	0.76	0.77	0.75
Dignity	0.64	0.08	1.29	1.33	0.45	0.66	0.08	1.29	1.33	0.49
ASCOT-Proxy-person (mean)	0.00	0.10	0.99	1.00		0.00	0.11	0.99	1.01	

*Abbreviations*: *Infit MNSQ* Information-weighted mean square, *Outfit MNSQ* Outlier-sensitive mean square

^a^Values in the range of .5 to 1.5 indicate a good fit

^b^Based on the category response curves we collapsed two domains (items): the lowest two (high-level of unmet needs, some needs) and highest two categories (no needs, ideal state) were combined for food and drink and for control over daily life.

**Table 7:** Item statistics including difficulty (in logits), infit and outfit mean square and point-measure correlations for the ASCOT-Carer

**Scale domain (item)**	**Item difficulty**	**SE**	**INFIT MNSQ**^**a**^	**OUTFIT MNSQ**^**a**^	**Point-measure correlation**
Personal safety	-3.23	0.14	1.20	**2.27**	0.46
Social participation and involvement	0.65	0.10	0.98	0.97	0.75
Control over daily life	0.33	0.11	0.91	0.91	0.75
Occupation	1.02	0.12	0.76	0.77	0.76
Feeling encouraged and supported	0.40	0.10	1.35	1.39	0.64
Space and time to be yourself	0.89	0.11	0.79	0.79	0.79
Self-care	-0.07	0.09	0.92	0.91	0.74
ASCOT-Carer (mean)	0.00	0.11	0.99	1.14	

*Abbreviations*: *Infit MNSQ* Information-weighted mean square, *Outfit MNSQ* Outlier-sensitive mean square.

^a^Values in the range of .5 to 1.5 indicate a good fit. Items highlighted in bold did not meet this criteria.

In this study, we kept the item in further analysis, as the item relates to an aspect of SCRQoL that has been identified as important to unpaid carers in previous research and is part of the ASCOT-Carer measure that has been widely used.

### Rasch analysis: Rating scale

As in another study [36], we evaluated the functionality of the ASCOT-Proxy-person and ASCOT-Carer 4-point rating scales using criteria proposed by Linacre: 1. the occurrence of more than 10 endorsements per response category, 2. the observation that both average measures and category thresholds increase across each response category, and 3. an observed OUTFIT MNSQ value of less than 2 for each response category [38]. If we observed disordered thresholds or group means or a high OUTFIT MNSQ for a category that had less than 10 respondents, we dismissed the finding (as we did not have enough evidence that subjects were unable to distinguish between the adjacent response categories).

Following domains (items) had observed average disordered in the ASCOT-Proxy-person (Table 8): food and drink; person cleanliness and comfort; and personal safety. In case of the ASCOT-Carer (Table 9), following items had OUTFIT MNSQ higher than two: personal safety; and feeling supported and encouraged.

**Table 8:** Response category (rating scale) diagnostics based on Linacre’s criteria: the ASCOT-Proxy-person

**Scale category**	**Observed count**	**% of counts**	**Observed average**	**OUTFIT MNSQ**	**Rasch-Andrich Threshold**	**Category measure**
**Food and drink**						
High-level of unmet needs	1	0	-0.10	1.00	NONE	-4.78
Some unmet needs	31	10	0.18	1.36	-2.27	-2.12
No unmet needs	63	21	0.71	1.43	1.04	-0.21
Ideal state	212	69	1.41	1.00	1.24	1.33
**Accommodation cleanliness and comfort**						
High-level of unmet needs	1	0	0.12	1.57	NONE	-4.15
Some unmet needs	13	4	**-0.20**	0.94	-1.39	-2.24
No unmet needs	61	20	0.51	0.92	0.23	-0.73
Ideal state	234	76	1.39	0.94	1.17	0.96
**Personal cleanliness and comfort**						
High-level of unmet needs	1	0	-0.10	1.25	NONE	-4.14
Some unmet needs	13	4	**-0.33**	0.83	-1.42	-2.28
No unmet needs	75	24	0.49	0.72	0.00	-0.67
Ideal state	222	71	1.46	0.93	1.41	1.19
**Personal safety**						
High-level of unmet needs	12	4	0.29	1.86	NONE	-2.49
Some unmet needs	38	12	**0.04**	0.81	-.84	-0.89
No unmet needs	76	25	0.95	1.12	0.17	0.28
Ideal state	182	59	1.51	1.06	0.67	1.76
**Social participation and involvement**						
High-level of unmet needs	67	22	0.24	0.97	NONE	-1.06
Some unmet needs	88	28	1.03	1.11	-0.89	0.53
No unmet needs	89	29	1.31	1.15	-0.06	1.81
Ideal state	66	21	2.03	1.69	0.95	3.45
**Occupation**						
High-level of unmet needs	81	26	0.19	0.79	NONE	-0.89
Some unmet needs	107	35	0.91	0.57	-1.14	0.84
No unmet needs	77	25	1.75	0.56	0.08	2.23
Ideal state	45	15	2.46	0.85	1.06	3.90
**Control over daily life**						
High-level of unmet needs	92	30	0.28	0.82	NONE	-0.59
Some unmet needs	73	24	0.85	0.86	-0.49	0.79
No unmet needs	78	25	1.59	0.64	-0.22	1.89
Ideal state	65	21	2.23	0.79	0.71	3.41
**Dignity**						
High-level of unmet needs	24	8	0.02	1.07	NONE	-2.18
Some unmet needs	86	30	0.85	1.56	-1.59	-0.23
No unmet needs	114	39	1.28	1.21	0.02	1.51
Ideal state	67	23	1.62	1.39	1.58	3.45

We evaluated the functionality of the ASCOT-Proxy-person 4-point rating scale using criteria proposed by Linacre: 1. the occurrence of more than 10 endorsements per response category, 2. the observation that both average measures and category thresholds increase across each response category, and 3. an observed OUTFIT MNSQ value of less than 2 for each response category [38]. Items highlighted in bold did not meet this criteria.

*Abbreviations*: *MNSQ* Mean square

**Table 9:** Response category (rating scale) diagnostics based on Linacre’s criteria: the ASCOT-Carer

**Scale category**	**Observed count**	**% of counts**	**Observed average**	**OUTFIT MNSQ**	**Rasch-Andrich Threshold**	**Category measure**
**Feeling encouraged and supported**						
High-level of unmet needs	35	11	-1.51	1.55	NONE	-3.51
Some unmet needs	122	39	-0.45	1.13	-2.76	-0.98
No unmet needs	114	36	1.42	0.95	0.03	1.80
Ideal state	42	13	1.92	**2.11**	2.74	4.28
**Space and time to be yourself**						
High-level of unmet needs	28	9	-2.03	0.92	NONE	-3.94
Some unmet needs	168	54	-0.33	0.70	-3.71	-0.78
No unmet needs	95	30	1.90	0.65	0.38	2.75
Ideal state	22	7	3.35	1.03	3.33	5.36
**Self-care**						
High-level of unmet needs	42	13	-2.10	0.66	NONE	-2.99
Some unmet needs	58	19	-0.71	0.79	-1.55	-1.32
No unmet needs	147	47	0.79	0.96	-0.92	0.79
Ideal state	66	21	2.25	1.17	2.47	3.53
**Personal safety**						
High-level of unmet needs	2	1	-4.16	0.62	NONE	-5.84
Some unmet needs	8	3	-2.11	1.45	-1.22	-4.22
No unmet needs	67	21	-0.72	**2.78**	-0.65	-2.49
Ideal state	235	75	0.87	1.22	1.87	-0.20
**Social participation and involvement**						
High-level of unmet needs	44	14	-1.58	1.12	NONE	-3.22
Some unmet needs	140	45	-0.23	0.94	-2.74	-0.55
No unmet needs	87	28	1.29	0.86	0.39	2.04
Ideal state	42	13	3.10	0.95	2.35	4.19
**Occupation**						
High-level of unmet needs	17	5	-1.98	1.00	NONE	-4.66
Some unmet needs	207	66	-0.21	0.78	-4.59	-0.81
No unmet needs	73	23	2.16	0.67	0.93	3.32
Ideal state	16	5	4.08	0.60	3.66	5.83
**Control over daily life**						
High-level of unmet needs	20	6	-2.06	1.00	NONE	-4.33
Some unmet needs	142	45	-0.58	0.80	-3.55	-1.34
No unmet needs	119	38	1.42	0.76	0.23	2.11
Ideal state	32	10	2.95	1.24	3.32	4.78

We evaluated the functionality of the ASCOT-Carer 4-point rating scale using criteria proposed by Linacre: 1. the occurrence of more than 10 endorsements per response category, 2. the observation that both average measures and category thresholds increase across each response category, and 3. an observed OUTFIT MNSQ value of less than 2 for each response category [38]. Items highlighted in bold did not meet this criteria.

*Abbreviations*: *MNSQ* Mean square

### Rasch analysis: Functionality of the response categories

**Fig. 5:**
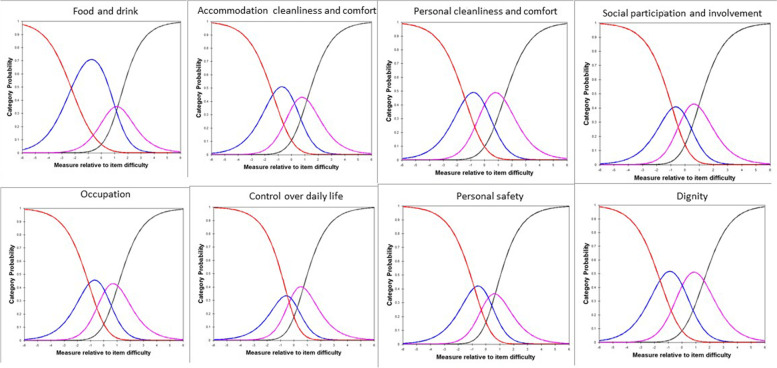
Category response curve for the ASCOT-Proxy-person. Note: Red: high-level of unmet needs; Blue: some unmet needs; Purple: no unmet needs; Black: ideal state

**Fig. 6:**
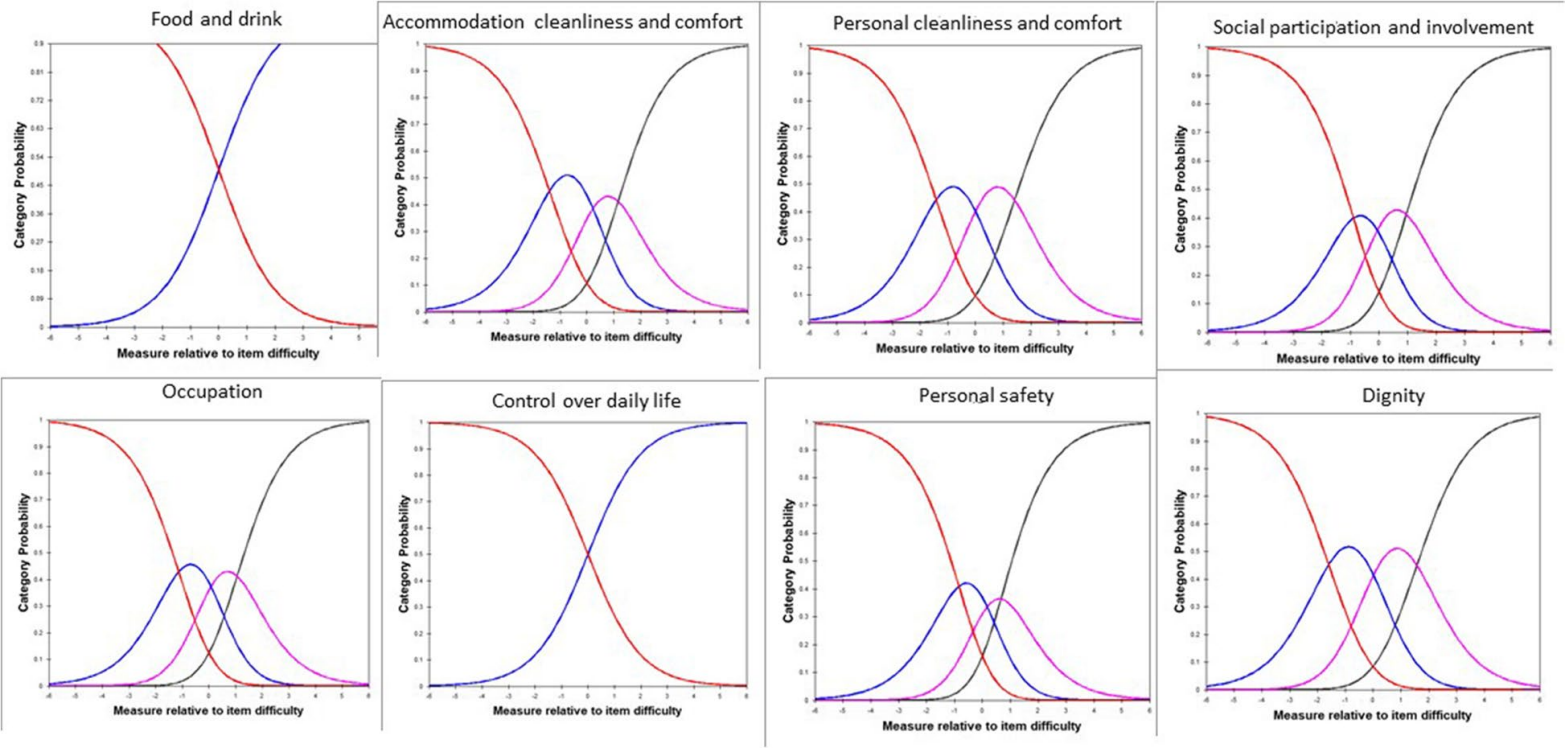
Category response curve for the ASCOT-Proxy-person: collapsed categories. Note: Red: high-level of unmet needs; Blue: some unmet needs; Purple: no unmet needs; Black: ideal state. In case of food and drink and control over daily life: Red: high-level of unmet needs and some unmet needs; Blue: no unmet needs and ideal state

We conducted Rasch analysis (the category probability curves) to examine whether unpaid carers are able to distinguish between the four responses options for each domain (item) (both the ASCOT-Proxy-person and the ASCOT-Carer). The category response curves indicated disordered thresholds for food and drink and control over daily life for the ASCOT-Proxy-person (Fig. 5). The lowest two (high-level of unmet needs, some needs) and highest two categories (no unmet needs, ideal state) were combined for food and drink. Similarly, the lowest two (high-level of unmet needs, some needs) categories and highest two categories (no unmet needs, ideal state) were combined for control over daily life (Fig. 6).

**Fig. 7:**
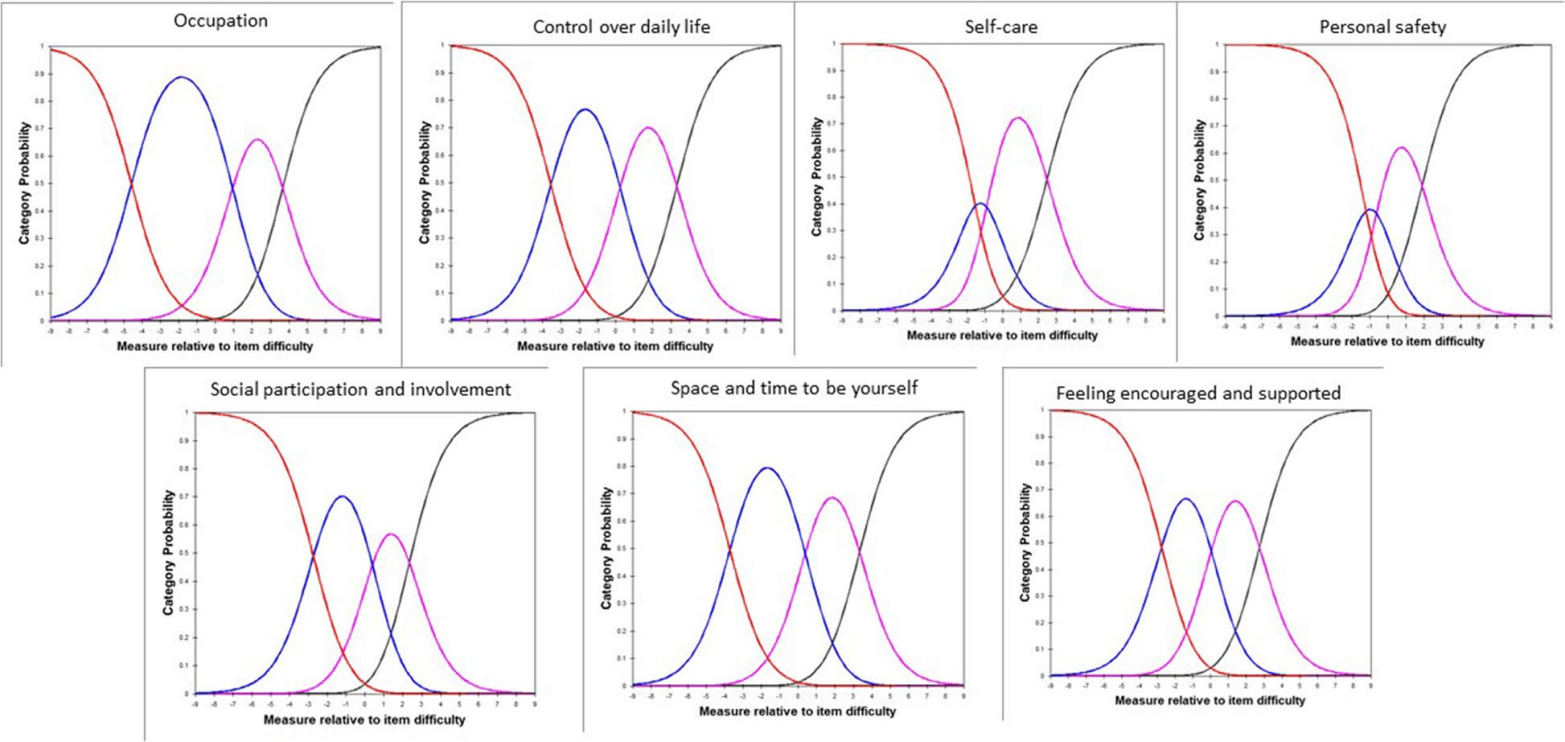
Category response curve for the ASCOT-Carer. Note: Red: high-level of unmet needs; Blue: some unmet needs; Purple: no unmet needs; Black: ideal state

There were no disordered category thresholds for the ASCOT-Carer (Fig. 7).

### Wright-Andrich maps: examination of floor and ceiling effects

**Fig. 8:**
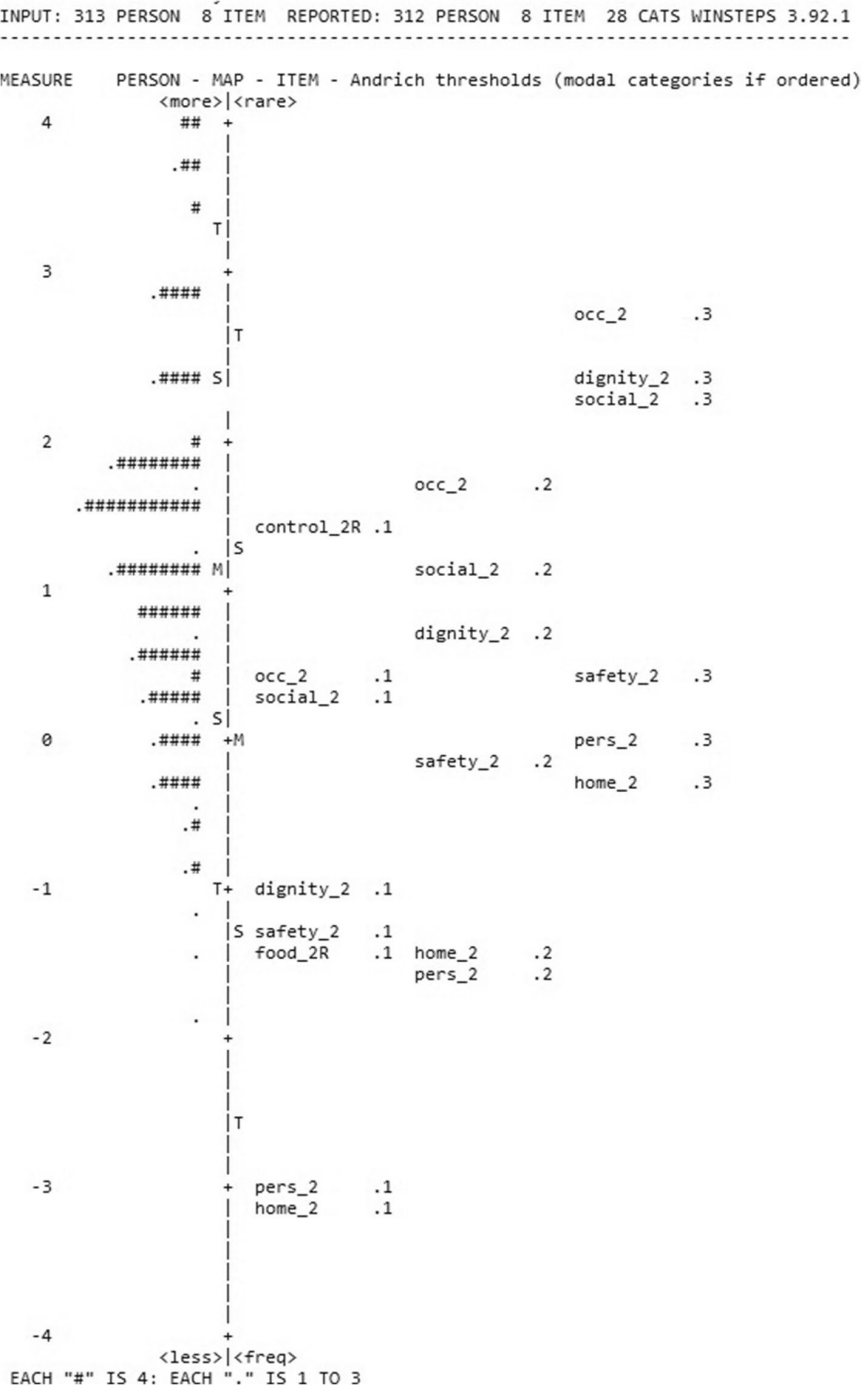
Results of Rasch Analysis, Wright-Andrich Map of the ASCOT-Proxy-person (collapsed categories). Note: The Measure Scale (-4 to +4) is the logit scale resulting from the Rasch Analysis. Top of the figure represents the floor (high-level of unmet needs coded as 0) while the bottom represents the ceiling (ideal state coded as 3) food and drink (collapsed; food_2R); accommodation cleanliness and comfort (home_2); personal cleanliness and comfort (pers_2); personal safety (safety_2); social participation and involvement (social_2); control over daily life (collapsed; control_2R); occupation (occ_2); dignity (dignity_2)

**Fig. 9:**
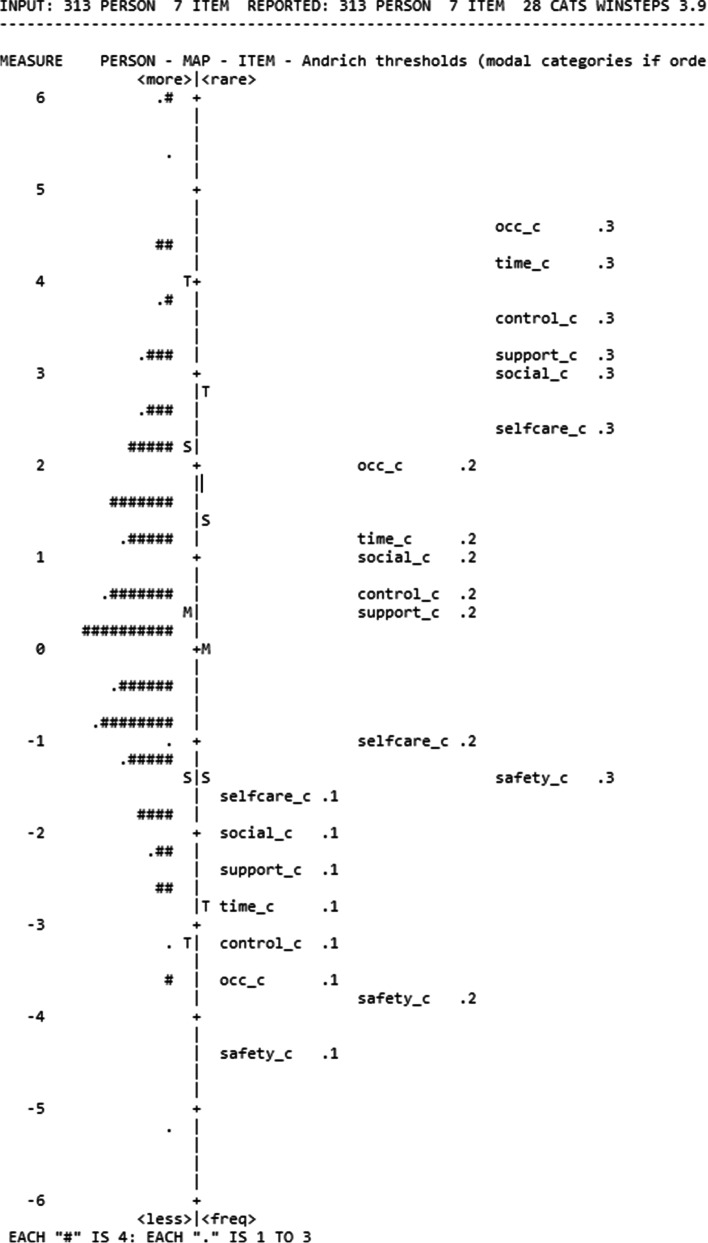
Results of Rasch Analysis, Wright-Andrich Map of the ASCOT-Carer. Note: The Measure Scale (-6 to +6) is the logit scale resulting from the Rasch Analysis. Top of the figure represents the floor (high-level of unmet needs coded as 0) while the bottom represents the ceiling (ideal state coded as 3) personal safety (safety_c); social participation and involvement (social_c); control over daily life (control_c); occupation (occ_c); feeling encouraged and supported (support_c); space and time to be yourself (time_c); self-care (selfcare_c)

Figures 8 and 9 present the Wright-Andrich Maps for the ASCOT-Proxy-person (collapsed categories for food and drink and control over daily life) and the ASCOT-Carer respectively. The Wright-Andrich Map plots the person measures along the left side and the item measures for each of the domains (items) along the right side. The M to the left of the centre line stands for the mean of the person logits. The M on the right represents the mean of the domain (item) logits. The S’s and T’s stand for 1 and 2 standard deviations from the means, respectively. From Fig. 8 (ASCOT-Proxy-person) we observed two significant gaps (approximately 1.5 logit) on the latent continuum. We see a lack of alignment between the person and item distributions, which is best characterised as the 1-point logit difference between the means of the items and persons. Similarly as in another study [36] floor and ceiling effects were defined as the existence of persons with logit scores at the bottom or top of the persons distribution (left side of the Wright-Andrich Map) that were at least one logit from the nearest item measure (right side of the Wright-Andrich Map). Specifically, we considered effects to be mild if less than 10% of respondents met this definition, moderate if 10% to 20% met the definition, and severe if more than 20% of respondents met the definition [36]. In case of the ASCOT-Proxy-person (Fig. 8), we detected a mild floor effect, which can be seen by the lack of overlap between persons and items at the top of the figure. Based on the Wright-Andrich Map, the ASCOT-Proxy-person would benefit from either adding new ‘easier’ items (within an existing cognitive framework) measuring slightly different aspects of the same domain or by moderating the intensity of the question using different wording.

From Fig. 9 (the ASCOT-Carer) we observed one significant gap (approximately more than 1 logit) on the latent continuum. We see that the two distributions are roughly aligned, (approximately less than .5 logit difference), as demonstrated by the proximity of the average person measure to the average item measure. In case of the ASCOT-Carer (Fig. 9) we detected a mild floor effect, which can be seen by the lack of overlap between persons and items at the top of the figure. Based on the Wright-Andrich Map, the ASCOT-Carer would benefit from either adding new ‘easier’ items (within an existing cognitive framework) measuring slightly different aspects of the same domain or by moderating the intensity of the question using different wording.’

### Rasch analysis: Differential Item Functioning

Lastly, we performed a Rasch differential item functioning (DIF) test to see if respondents conceptualised items differently based on the mode of administration (postal versus online). In the analysis of uniform DIF by survey administration, the Mantel-Haenszel statistic, adjusted for multiple comparisons (*p*<0.007), indicated DIF for two items from the ASCOT-Carer: occupation (χ^2^=12.33, *p*=0.0004) and feeling encouraged and supported (χ^2^=14.33, *p*=0.0002). This indicates that these two items respond differently, by survey administration group. For occupation, the DIF measure was 1.71 (DIF S.E. =0.30) for those who completed the survey via postal return and 0.87 (DIF S.E. = 0.14) for those who completed the survey online (DIF contrast=0.84, joint S.E. =0.33). For feeling encouraged and supported, the DIF measure was -0.47 (DIF S.E. =0.22) for those who completed the survey via postal return and 0.61 (DIF S.E. = 0.11) for those who completed the survey online (DIF contrast=-1.08, joint S.E. =0.25).

To better understand why respondents conceptualised items differently based on the mode of administration (postal versus online), we have explored whether the two samples (postal and online survey) differed in sociodemographic characteristics: age, gender and ethnicity. We identified that those who filled in postal version were older than those who filled in online version (mean age: 70.63 versus 60.38 years). Therefore, we performed a Rasch DIF test to see if respondents conceptualised items differently based on their age (under 65 years old versus 65 years or over). In the analysis of uniform DIF by age, the Mantel-Haenszel statistic, adjusted for multiple comparisons (*p*<0.007), indicated DIF for two items from the ASCOT-Carer: occupation (χ^2^=13.53, *p*=0.0002) and feeling supported and encouraged (χ^2^=12.73, *p*=0.0004). For the occupation, the DIF measure was 0.72 (DIF S.E. =0.16) for those who were under 65 years old and 1.44 (DIF S.E. =0.20) for those 65 years or over (DIF contrast=-0.72, joint S.E.=0.25). For feeling encouraged and supported, the DIF measure was 0.77 (DIF S.E. =0.13) for those who were under 65 years old and -0.06 (DIF S.E. =0.15) for those 65 years or over (DIF contrast=0.83, joint S.E.=0.20).

There was no evidence of DIF by survey administration or age for the ASCOT-Proxy-person (*p*>.006).

### Reliability/Internal consistency

The Cronbach’s alpha was 0.69 for the ASCOT-Proxy-person; and 0.83 for the ASCOT-Carer. The ordinal alpha was 0.78 for the ASCOT-Proxy-person; and 0.87 for the ASCOT-Carer, which meets the .70 reliability standard [39].

### Construct validity

Table 1 in Additional File 2 provides an overview of the hypotheses used in this study for assessing construct validity. Table 10 shows Spearman correlation coefficients between the ASCOT-Proxy-person, ASCOT-Carer overall scores (continuous variables) and related constructs (continuous variables). The majority of the Spearman’s rank correlations (>75%) between the ASCOT-Proxy-person, ASCOT-Carer and other variables were same as hypothesised. There were few exceptions to this. The correlations between ASCOT-Proxy-person overall scores and DEMQOL-Proxy-proxy were weaker than expected but stronger with I/ADLs (Proxy-proxy) than expected.

**Table 10:** Convergent validity of the ASCOT-Proxy-person and ASCOT-Carer

	**ASCOT-Proxy-person**			**ASCOT-Carer**		
**Variables**	**N**	**Rs**	**As hypothesised**	**N**	**Rs**	**As hypothesised**
**Well-Being & Health**						
EQ-5D-5L (carer)	n/a	n/a	n/a	312	0.42***	**YES**
EQ-5D-5L-Proxy-proxy	278	0.38***	**YES**	n/a	n/a	n/a
EQ-5D-5L-Proxy-person	275	0.41***	**YES**	n/a	n/a	n/a
C-DEMQOL	n/a	n/a	n/a	303	0.75***	**YES**
DEMQOL-Proxy-proxy	268	0.44***	Exp: large positive correlation This study: medium positive	n/a	n/a	n/a
Carers quality of life (one item)	n/a	n/a	n/a	311	0.71***	**YES**
Proxy-proxy quality of life (one item)	276	0.40***	**YES**	n/a	n/a	n/a
Proxy-person quality of life (one item)	277	0.61***	**YES**	n/a	n/a	n/a
Carer Experience Scale	n/a	n/a	n/a	308	0.65***	**YES**
ASCOT-Proxy-proxy	n/a	n/a	n/a	283	0.10	Exp: small positive correlation This study: small positive correlation, ns
ASCOT-Proxy-person	n/a	n/a	n/a	277	0.18*	**YES**
Satisfaction with social care services	n/a	n/a	n/a	308	0.40***	**YES**
**Sociodemographic**						
(I/ADLS): proxy-proxy^a^	276	-0.30***	Exp: small negative correlation This study: medium negative	n/a	n/a	n/a

We used the correlation coefficient as a measure of the size of the effect. We interpreted values of ± 0.1 as a small effect, ± 0.3 as a medium effect and ± 0.5 as a large effect Bold indicates that the magnitude and direction of the correlation was as hypothesised

*Abbreviations*: *Rs* The spearman correlation coefficient, *n/a* Not used for validation, *Exp* Expected, *ns* Non-significant

^a^Instrumental activities of daily living (I/ADLS): total number of eight ADLs with difficulty or unable to complete alone (higher the score, the more ADLs with difficulty: getting around (except steps) indoors; getting in and out of bed; eating; paperwork or finances; having a bath or shower; dressing or undressing; using the toilet; washing hands and face)

^*^< 0.05; ^**^< 0.01; ^***^< 0.001

Table 11 displays the associations between the ASCOT-Proxy-person, ASCOT-Carer overall scores and various subgroups of the sample. Both measures detected differences between their scores and various subgroups as hypothesised.

**Table 11:** Known-groups validity of the ASCOT-Proxy perspectives and ASCOT-Carer

	**ASCOT-Proxy-person**			**ASCOT-Carer**		
**Variables**	**N**	**Median**	**As hypothesised**	**N**	**Median**	**As hypothesised**
**Home design suitability** proxy-proxy	278	H(3)= 13.747**	**YES**	312	H(3)= 18.649***	**YES**
Meets their needs very well	89	0.71		101	0.70	
Meets most of their needs	132	0.66		146	0.66	
Meets some of their needs	47	0.64		55	0.54	
Totally inappropriate	10	0.40		10	0.57	
**Impact of caring on health**				300	H(1)= 13.758***	**YES**
Yes	n/a	n/a	n/a	285	0.66	
No	n/a	n/a	n/a	15	0.92	
**Caregiving situation**						
Lives in the same household				312	H(1)= 38.378***	**YES**
Yes	n/a	n/a	n/a	180	0.75	
No	n/a	n/a	n/a	132	0.60	
Relationship				312	H(4)= 19.440***	**YES**
Spouse/partner	n/a	n/a	n/a	129	0.61	
Parent	n/a	n/a	n/a	153	0.70	
Sibling	n/a	n/a	n/a	4	0.89	
Child	n/a	n/a	n/a	1	0.66	
Other	n/a	n/a	n/a	25	0.83	
Hours of care per week				309	H(4)= 68.137***	**YES**
0-9	n/a	n/a	n/a	48	.85	
10-19	n/a	n/a	n/a	47	.73	
20-34	n/a	n/a	n/a	36	.71	
35-49	n/a	n/a	n/a	32	.56	
50 or more	n/a	n/a	n/a	146	.60	
Help with personal care				310	H(1)= 30.446***	**YES**
Yes	n/a	n/a	n/a	230	0.62	
No	n/a	n/a	n/a	80	0.73	
Help with medicines				310	H(1)= 30.838***	**YES**
Yes	n/a	n/a	n/a	251	0.65	
No	n/a	n/a	n/a	59	0.85	
**Sociodemographic characteristics**						
Carer’s gender				312	**H(1)= 1.655**	**YES**
Female	n/a	n/a	n/a	237	0.66	
Male	n/a	n/a	n/a	75	0.67	
Carer in paid employment				312	H(1)= 7.431**	**YES**
Yes: full- or part-time	n/a	n/a	n/a	120	0.71	
No	n/a	n/a	n/a	192	0.66	

Bold indicates that the group differences were as hypothesised.

*H* Chi-squared with ties including d.f., *n/a* Not used for validation, *Exp*. Expected

^*^< 0.05; ^**^< 0.01; ^***^< 0.001

## Discussion

The aim of this cross-sectional study where data were collected using self-administered questionnaire (online or paper form) was to establish the feasibility, construct validity and reliability of the ASCOT-Proxy and ASCOT-Carer among unpaid carers of people with dementia who are unable to self-report. In addition, we aimed to compare the structural characteristics of the ASCOT-Proxy (an adapted version of the ASCOT-SCT4), against the original ASCOT-SCT4 [10].

Overall, the percentage of missing values was low for the ASCOT-Proxy (both perspectives) and ASCOT-Carer indicating a good acceptance of both measures among unpaid carers of people with dementia who are unable to self-report. It is important to mention though, that the dignity item had a higher percentage of missing data in the ASCOT-Proxy (Proxy-proxy: 6.71%; Proxy-person: 7.03%). The overall scores for SCRQoL are only calculated when all items have non-missing values [10]. As such missing data imputation techniques should be considered when calculating overall SCRQoL score.

Next, we found that the proportion of missing data was significantly higher for participants who used paper questionnaires when compared to those using online version. This should not be interpreted that online version of the ASCOT-Proxy was more acceptable than the paper one. For example, unlike the paper version, where participants could accidentally skip over pages, the online version asked participants if they wished to leave the question blank. In addition, when participants could choose which form they wanted to fill in those who were older preferred the paper version (70.6 vs 60.4 years old). Without being able to offer the paper version, it is possible participants would not be willing to take part. This was highlighted to us by research staff across NHS sites, who recruited approximately half of the participants (140/313) on our behalf. Of these, 55 people recruited through the NHS chose to fill in a paper questionnaire, as opposed to 88 people who selected online questionnaire. Only eight participants out of 159 recruited through JDR selected a paper form. It is important to know that those recruited through this platform for the majority of time (between April and October 2020) did not have an option to fill in paper form of the questionnaire due to lack of access to printing facilities by research team as a result of lockdown measures [27]. This is important, as it highlights that there are issues with exclusion of some participants if offering only an online option as a mode for data collection.

In this study, we tested the structural characteristics of the ASCOT-Proxy using exploratory factor analysis against the ASCOT-SCT4 [10]. We confirmed a weak unidimensional (one-factor) solution for the ASCOT-Proxy-person. Similar to this study, one-factor structure has been found for the ASCOT-SCT4 in samples of older adults [10] as well as for other adapted version, e.g. easy-read ASCOT [13] and the translation of the ASCOT into Finnish [35]. On the other hand, we found two factor solution for the ASCOT-Proxy-proxy representing basic domains (items) (Factor 1) and higher order domains (items) Factor 2 [10]. As highlighted in the Results section, the two factor solution for the ASCOT-Proxy-proxy was, while robust, a novel finding. Therefore, future studies should report the factor structure for ASCOT-Proxy-proxy and if our results are replicated, it has to be considered carefully whether and how the ASCOT-Proxy-proxy tool should be used (e.g. in terms of calculating overall scores, establishing construct validity etc.). It is important to highlight though, that the ASCOT-Proxy has been developed and tested at all stages of its development with both perspectives, including this study, so we do not know how e.g. the ASCOT-Proxy-person would perform on its own. More importantly, inclusion of both perspectives improved the acceptability and face validity of the measure during earlier stages of its development [21].

Next, the dignity domain (item) had a low factor loading in case of both ASCOT-Proxy perspectives. In this study, as explained in the Results section (Factor structure of ASCOT-Proxy perspectives) we retained dignity domain (item) as ASCOT-Proxy was developed to conceptually align closely to the ASCOT-SCT4 self-completion tool where each domain (item) represents one dimension of SCRQoL. Dignity is an important aspect of SCRQoL as established in ASCOT-SCT4 development studies [10] and the item was acceptable when developing the ASCOT-Proxy [21]. However, as this was first study to explore structural characteristics of the ASCOT-Proxy, it is important to test structural characteristics of the ASCOT-Proxy (both perspectives) as part of future studies to see whether there are any issues with the dignity item. If our findings are replicated it may be necessary to revisit whether dignity should be included as an important aspect of SCRQoL of social care users when the answers are provided by proxy (e.g. through qualitative conceptual studies with unpaid carers).

In addition to evaluating psychometric properties of the ASCOT-Proxy and ASCOT-Carer by Classical Test Theory methods, we have conducted Rasch analysis [40, 41] to further evaluate the measurement properties of the ASCOT-Proxy-person and ASCOT-Carer. To summarise, the following issues were highlighted when conducting Rasch analysis. First, the ASCOT-Carer personal safety was outside of OUTFIT MNSQ range of .5 to 1.5 when evaluating overall model fit. This is the first study to suggest there may be an issue with the personal safety item in the ASCOT-Carer. It is important to keep in mind though that this was the first time when psychometric characteristics of the ASCOT-Carer were tested in a sample of unpaid carers of someone living with dementia in England. This was also the first time when the Rasch analysis was used as an approach when investigating psychometric characteristics of the ASCOT-Carer. It is also important to highlight that we collected data during COVID-19 pandemic which could impact how people responded when asked about their personal safety. Therefore, we suggest when the ASCOT-Carer is going to be used in the future as a measure, researchers will explore whether there are any issues with personal safety item as identified in this study. Next, this study indicates that respondents may not distinguish between all four response categories for food and drink and control over daily life in case of the ASCOT-Proxy-person. There is an indication that two-response categories option (by combining the two lowest categories and the two highest categories) may be a better solution than four response categories for both food and drink and control over daily life. Next, based on Wright-Andrich maps we detected a mild floor effect in case of both ASCOT-Proxy-person and ASCOT-Carer. Based on the Wright-Andrich Map, both measures would benefit from either adding new ‘easier’ items (within an existing cognitive framework) measuring slightly different aspects of the same domain or by moderating the intensity of the question using different wording. Lastly, Rasch differential item functioning test indicated that the differential item functioning in case of two items in the ASCOT-Carer (occupation; feeling supported and encouraged) when compared postal vs online form of survey is not a bias of the tool but it is likely due to differences between the two samples, i.e. those who filled in postal version were older (70.63 years old) than those who filled in online version (60.38 years old). This implies that the samples cannot be readily compared, unless the items are removed or different item measures are used to score subjects based on their age. The latter would require using Rasch person measures for calculating the preference-weighted values. This is something what should be explored in the future.

Looking across the results when testing convergent and known-group validity, we conclude that both ASCOT-Proxy-person and ASCOT-Carer are valid measures of SCRQoL. Both ASCOT-Proxy-person and ASCOT-Carer met the .70 reliability standard [39] when we calculated ordinal alpha (introduced by Zumbo et al. [42]). The .70 reliability standard was not met when we calculated Cronbach’s alpha for ASCOT-Proxy-person, however it is known that Cronbach’s alpha underestimates the reliability of the ordinal response scales [43]. It is important to highlight that in the present study, we only investigated one aspect of reliability (internal consistency) while test-retest reliability has not been studied. This was due to the impact of COVID-19 on this study, as we only managed to collect data at one time point. Therefore, test-retest reliability should be established in the future studies.

The most important potential limitation of our study is the lack of access to information about the characteristics of participants who started but did not finish the questionnaire (*n*=33) as we did not have permission to use information from incomplete online survey. Having the access to this information, would allow us to understand whether the questionnaires are more feasible for some participants. We were also unable to recruit participants through the providers of adult social care including local authorities, independent sector care providers and voluntary sector organisations potentially introducing selection bias in our sample. Instead, all our participants were recruited through the NHS and JDR. In this study, we used the same preference-weighted values for ASCOT-Proxy, as for ASCOT-SCT4. A subsequent step should be the development of preference weights for ASCOT-Proxy, which represent the relative importance of the response levels of each domain for quality of life. For example, there is suggestion, that self-reported measures used by people with dementia and proxy versions need different preference-weight values [44]. Next, we did not compare self- and proxy-report. This was not possible in this study, as we focussed on those unable to self-report (our main target group).

## Conclusions

This was a first study to explore psychometric characteristics of the ASCOT-Proxy and ASCOT-Carer, with unpaid carers of people with dementia living at home unable to self-report. There are some aspects of the psychometric characteristics of the ASCOT-Proxy and ASCOT-Carer that warrant further investigation in future work.

## Supplementary Information

**Additional file 1:** List of measures used for establishing construct validity of ASCOT-Proxy and ASCOT-Carer.

**Additional file 2:** Statistical analysis

## Data Availability

Not available.

## Abbreviations

ASCOT: The Adult Social Care Outcomes Toolkit
ASCOT-Carer: the carer version of ASCOT
ASCOT-Proxy: a proxy-report version of ASCOT
I/ADLs: Instrumental activities of daily living and activities of daily living
Infit MNSQ: Information-weighted mean square
JDR: Join Dementia Research
MNSQ: Mean square
NHS: the National Health Service
Outfit MNSQ: Outlier-sensitive mean square
SCRQoL: Social Care-Related Quality of Life
UK: the United Kingdom
