# Supplementary material for: Feasibility, validity and reliability of the ASCOT-Proxy and ASCOT-Carer among unpaid carers of people living with dementia in England
Source: Health Qual Life Outcomes. 2023 Jun 3;21:54. doi: 10.1186/s12955-023-02122-0 (PMC10239280; doi:10.1186/s12955-023-02122-0)
Supplement: Supplementary file 1 — Additional file 1: List of measures used for establishing construct validity of ASCOT-Proxy and ASCOT-Carer. [file 12955_2023_2122_MOESM1_ESM.docx]

**Additional file 1**

***List of measures used for establishing construct validity of ASCOT-Proxy and ASCOT-Carer***

European Quality of Life-5 Dimensions (EQ-5D) questionnaires: the EQ-5D questionnaires are a standardised measure of current health (today) used in clinical and economic appraisals among healthy populations as well as among cohorts with a range of diseases [1]. In the present study we used three versions of the questionnaire: EQ-5D-5L to measure carer’s self-rated health-related quality of life and EQ-5D-5L-Proxy version 1 (proxy-proxy) and version 2 (proxy-person). Each EQ-5D instrument included five items measuring five dimensions of health-related quality of life: mobility, self-care, usual activities (e.g. work, study, housework etc.), pain/discomfort and anxiety/depression. Each item had five response options: no problems, slight problems, moderate problems, severe problems, unable to/extreme problems [1]. We calculated a single health state index score from individual health profiles using the EQ-5D-5L crosswalk value sets (UK region) developed by the EuroQol Group (version 2) [2]. The score can range from less than 0 (where 0 is the value of a health state equivalent to dead; negative values representing values as worse than dead) to 1 (the value of full health), with higher scores indicating higher health utility [1].

C-DEMQOL (self-administered version): measures carer quality of life for family carers of people with dementia. It includes 30 questions subdivided under five categories including feeling supported, carer patient relationship, meeting personal needs, confidence in the future and carer wellbeing [3]. We calculated the overall score following published syntax [4]. Higher scores indicate higher quality of life. We did not impute missing values.

DEMQOL-Proxy: is a 31-item interview questionnaire answered by a carer measuring health-related quality of life of people with dementia [5]. Carers are asked about the person’s feelings, memory and everyday life in last week and are encouraged to think about what they think the person would answer (proxy-proxy perspective). In this study, the measure was included in the self-completion questionnaires, rather than administered as an interview. We calculated an overall score [6]. Higher scores indicate higher quality of life. We did not impute missing values and carers filled in the questionnaire themselves as part of the survey.

Overall quality of life: was measured using one item asking carers about their overall quality of life, including e.g. standard of living, surroundings etc. Carers could choose from seven response options ranging from very good (1), through neither good nor bad (3) to very bad (5). We asked carers the same question about the person living with dementia (both proxy-proxy and proxy-person perspectives).

The Carer Experience Scale (CES): is a profile measure of caring experience. It includes six attributes of care-related quality of life that are important to unpaid carers: activities, support (from family and friends), assistance (from organisations), fulfilment, control, and getting-on with the care recipient [7]. We calculated a preference-based overall score of caring experiences (0 = bottom state, 100 = top state) following the published formula [8].

Satisfaction with social care services [9]: carers were asked ‘overall, how satisfied or dissatisfied are you with the support or services you and the person you care for have received in the last 12 months’. Carers could choose from ‘I am extremely satisfied’ to ‘I am extremely dissatisfied’ (7-point Likert scale, the higher the score, the more satisfied with the services).

Home design suitability for care recipient’s needs: carers were asked whether in their opinion (proxy-proxy perspective), the person they care for’s home is designed to meet their needs. Participants could choose from: meets their needs very well (1), meets most of their needs (2), meets some of their needs (3), and totally inappropriate (4).

Impact of caring on health as used in the Survey of Carers in Households – England 2009/10 [10]: carers were asked whether their own health had been affected by the care they provide and could choose from different options (e.g. feeling tired; feeling depressed; loss of appetite; disturbed sleep; physical strain etc.). For the purpose of this paper, this variable was categorised into: no, impact on health/impact on health).

Caregiving situation: whether the carer and care-recipient lives in the same household, relationship to a person living with dementia, estimated hours of care per week, whether the carer provides help with personal care or medicines. These items were used in the Survey of Carers in Households - England, 2009-10 [11].

Sociodemographic characteristics: included age (carer and person with dementia); gender (carer); ethnicity (carer); region (carer); and instrumental activities of daily living and activities of daily living (I/ADLS) (proxy-proxy), adapted from questions in the Adult Social Care Survey [12].

Mode of data collection: paper questionnaire or online.

Both online survey and paper version of the questionnaires included the same questions, presented in the same order with the exception of a visual analogue scale (EQ VAS) [13] that was only included in the online survey. First, we piloted the online survey and paper-based version of questionnaires with two patient and public involvement (PPI) members and one member of Personal Social Services Research Unit, University of Kent, not involved in the ‘Measuring Outcomes of People with Dementia and their carers’ (MOPED) study. We then piloted the online survey from 27th January 2020 until 27th February 2020 with 19 carers living in Kent, Sussex, Surrey and South of London recruited through the Join Dementia Research (JDR). The data from this pilot were included in the present analysis.

**References**

1. Foundation., E.R., *EQ-5D-5L User Guide Version 3.0 September 2019*. 2019.

2. van Hout, B., et al., *Interim scoring for the EQ-5D-5L: mapping the EQ-5D-5L to EQ-5D-3L value sets.* Value Health, 2012. **15**(5): p. 708-15.

3. *C-DEMQOL - a measure of carer quality of life for carers of someone with dementia.* 30/09/2019]; Available from: <https://www.bsms.ac.uk/research/neuroscience/cds/research/cdemqol.aspx>.

4. *SPSS SYNTAX FILE FOR CODING C-DEMQOL DATA – JUNE 2018**. 2018 [cited 2021 04/08/2021]; Available from: <https://www.bsms.ac.uk/_pdf/cds/spss-syntax-for-c-demqol-final.pdf>.

5. Smith, S.C., et al., *Development of a new measure of health-related quality of life for people with dementia: DEMQOL.* Psychol Med, 2007. **37**(5): p. 737-46.

6. *SPSS SYNTAX FILE FOR RECODING DEMQOL-PROXY DATA - JUNE 2005*. [cited 2021 04/08/2021]; Available from: <https://www.bsms.ac.uk/_pdf/cds/spss-syntax-demqol-proxy.pdf>.

7. *The Carer Experience Scale (CES)*. 30/09/2019]; Available from: <https://www.birmingham.ac.uk/research/activity/mds/projects/HaPS/HE/ICECAP/CES/index.aspx>.

8. Al-Janabi, H., T.N. Flynn, and J. Coast, *Estimation of a preference-based carer experience scale.* Med Decis Making, 2011. **31**(3): p. 458-68.

9. Department of Health and Social Care, *Carers strategy: the second national action plan 2014 to 2016.* 2014: London.

10. Fox, D., Holder, J., Netten, A. , *Personal Social Services Survey of Adult Carers in England – 2009-10: Survey Development Project. Technical Report.* 2010, Personal Social Services Research Unit, University of Kent: Canterbury.

11. NHS Digital. *Survey of Carers in Households - England, 2009-10*. 2010 27/09/2019]; Available from: <https://digital.nhs.uk/data-and-information/publications/statistical/survey-of-carers-in-households/survey-of-carers-in-households-england-2009-10>.

12. Malley, J., Caiels, J., Fox, D., McCarthy, M., Smith, N., Beadle-Brown, J., Netten, A., Towers, A-M., *A report on the developmental studies for the National Adult Social Care User Experience Survey* 2010, Personal Social Services Research Unit.

13. *EQ-5D*. 25/09/2019]; Available from: <https://euroqol.org/>.
