# Supplementary material for: Feasibility, validity and reliability of the ASCOT-Proxy and ASCOT-Carer among unpaid carers of people living with dementia in England
Source: Health Qual Life Outcomes. 2023 Jun 3;21:54. doi: 10.1186/s12955-023-02122-0 (PMC10239280; doi:10.1186/s12955-023-02122-0)
Supplement: Supplementary file 2 — Additional file 2: Statistical analysis [file 12955_2023_2122_MOESM2_ESM.docx]

**Additional file 2**

***Statistical analysis***

Descriptive analysis

We summarised normally distributed continuous variables as means and standard deviations (SD), skewed continuous variables as medians and interquartile range (IQR), and categorical variables as the number and percentage of participants within each category.

Feasibility

We evaluated the feasibility of the ASCOT-Proxy (both perspectives) and ASCOT-Carer by estimating the proportion of missing data and whether this differed by the mode of data collection.

Structural characteristics of the ASCOT-Proxy

The ASCOT-Proxy is an adapted version of the ASCOT-SCT4, which has been found to have a weak unidimensional structure using exploratory factor analysis and Mokken scale analysis [1]. The original developers noted that weak unidimensionality is desirable, since the ASCOT is a preference-based measure. Indeed, some have argued that the ASCOT (like the EQ-5D [2] and ICECAP-O [3]) ought to be understood as a formative measure, so it is not appropriate to apply exploratory factor analysis [1] (see pp.113-115). Indeed, there is some evidence that the ASCOT may be best understood as a formative/mixed, formative and reflective, measure [4]. Nevertheless, the testing of structural characteristics may be useful in the development of adapted or translated versions of the measure. In this study, the aim was to test the structural characteristics of the ASCOT-Proxy using exploratory factor analysis against the ASCOT-SCT4.

As the ASCOT-Proxy has Likert-type items which yield ordinal data, we undertook several steps when examining structural characteristics of the ASCOT-Proxy (both perspectives) as recommended by Gugiu et al. [5].

First, we conducted Horn’s parallel analysis to determine the number of factors to retain. To do this, we applied user-written command ('paran') by Alexis Dinno [6]. We used principal component analysis (pca) as a factor estimation type. We generated 5000 random correlation matrixes for analysis, using 95^th^ percentile for randomly generated eigenvalues similarly as in a previous study [7]. To inform our decision regarding the number of factors to retain, we compared the observed principal component eigenvalues with the 95^th^ percentile eigenvalues (random) from the simulated datasets. We retained those factors (components) where observed eigenvalues exceeded the eigenvalues generated by random.

Next, we performed exploratory factor analysis on the polychoric correlation matrix (ordinal exploratory factor analysis). More specifically, we used user-written command (‘polychoric’) by Stas Kolenikov v1.4 to generate a matrix of polychoric correlations. Then, we used the ‘matrix’ command to store the polychoric correlation matrix, so that we could use it with the ‘factormat’ command to perform maximum likelihood exploratory factor analysis. We specified to retain the number of factors as suggested by Horn’s parallel analysis.

Next, we verified that the suggested solution from the parallel analysis, supported the stability and interpretability of the factors structure. We also checked whether any items had low factor loadings (<.4) on all the factors, or salient loadings (>.5) on two factors [5].

Factor structure of the ASCOT-Carer using the same data as this study has been established elsewhere and, hence, is not included in this paper [4].

Rasch analysis

As Rasch analysis overcomes some of the issues identified with the use of the Classical Test Theory methods [8, 9], we applied Rasch analysis to gain further insight into psychometric properties of the ASCOT-Proxy and ASCOT-Carer. Study by Shi et al. 2019 (Table 1 [10]) provides an overview of item statistics generated from Rasch analysis, their definitions and guide for interpretation.

Rasch analysis is a logit modelling technique that converts the ordinal scores for the scale items into a continuous latent scale (log-odd units), with individual responses positioned onto that scale. It is an accepted method of evaluating quality of life instruments [11, 12]. Rasch analysis was conducted in WINSTEPS software (version 3.92.1.). An example of steps that we followed in WINSTEPS for the ASCOT-Carer is provided at the end of this document (we applied similar steps for the ASCOT-Proxy).

We applied a polytomous version of the Rasch model as the ASCOT-Carer and ASCOT-Proxy domains (items) have four response options per each domain (item). We selected a partial credit model [13] since the distance between thresholds is not assumed to be equal across domains (items) [12]. More specifically, while the response options correspond to four different outcome states (ideal state, no unmet needs, some unmet needs, and high-level of unmet needs) their wording differs for each domain (item). For example in the ASCOT-Proxy for food and drink, the response options are: ‘As clean and comfortable as s/he wants’; ‘Adequately clean and comfortable’; ‘Not quite clean or comfortable enough’, ‘Not at all clean or comfortable’. For occupation, the response options are: ‘Is able to spend his/her time as s/he wants, doing the things s/he values or enjoys’; ‘Is able to do enough of the things s/he values or enjoys with his/her time’; ‘Does some of the things s/he values or enjoys with his/her time, but not enough’; ‘Doesn’t do anything s/he values or enjoys with his/her time’. For the ASCOT-Carer the response options for occupation are: ‘I’m able to spend my time as I want, doing things I value or enjoy’; ‘I’m able to do enough of the things I value or enjoy with my time’; ‘I do some of the things I value or enjoy with my time, but not enough’; ‘I don’t do anything I value or enjoy with my time’. For self-care the response options are: ‘I look after myself as well as I want’; ‘I look after myself well enough’; ‘Sometimes I can’t look after myself well enough’; ‘I feel I am neglecting myself’.

Rasch analysis: Overall model fit

We assessed model fit using the information-weighted mean square (INFIT MNSQ), outlier-sensitive mean square (OUTFIT MNSQ) and point-measure correlation. Similarly as in another study [10], we considered values of INFIT and OUTFIT MNSQ statistics in the range of .5 to 1.5 as satisfactory [14]. As highlighted by Shi et al. [10] good fitting items should exhibit a positive correlation with the subscale formed by the Rasch analysis (point-measure or item-Rasch measure correlation). A negative point-measure correlation is an indication that response categories have been reversed (i.e., lower response categories have a higher item measure than subsequent response categories).

Rasch analysis: Rating scale

As in another study [10], we evaluated the functionality of the ASCOT-Proxy and ASCOT-Carer 4-point rating scales using criteria proposed by Linacre: 1. the occurrence of more than 10 endorsements per response category, 2. the observation that both average measures and category thresholds increase across each response category, and 3. an observed OUTFIT MNSQ value of less than 2 for each response category [15].

Rasch analysis: Functionality of the response categories

We conducted a Rasch analysis (the category probability curves) to examine whether unpaid carers are able to distinguish between the four responses options for each domain (item) (both the ASCOT-Proxy and ASCOT-Carer). The category probability curves show the likelihood that a subject with a specific person measure relative to item difficulty will select the category [16]. The threshold is the midpoint between adjacent response categories so that it reveals the point where the likelihood of choosing either response category is the same [17]. If the right number of response options are selected, the category calibration increases in an orderly manner. If a disordered threshold occurs, the category needs to be collapsed into an adjacent category [16]

Wright-Andrich maps: examination of floor and ceiling effects

We used a graphical method (Wright-Andrich maps, Table 1.7 in WINSTEPS) to plot the distributions of item and person measures for the ASCOT-Proxy and ASCOT-Carer. Ideally, the distribution of person measures aligns with the distribution of item measures (e.g. means should be about the same, range should be similar). Gaps in measurement occur when the person’s and item’s distributions do not align. Gaps indicate a lack of precision in measurement at that place on the scale. Gaps can appear anywhere along the latent continuum. A floor effect is a region at the bottom of the scale where there were a significant number of person measures but no item measures nearby (not enough easy items to cover the bottom of the person distribution). A ceiling effect is a region at the top of the scale where there were a significant number of person measures but no item measures nearby (not enough difficult items to cover the top of the person distribution). Specifically, we considered effects to be mild if less than 10% of respondents met this definition, moderate if 10% to 20% met the definition, and severe if more than 20% of respondents met the definition [10].

Rasch analysis: Differential Item Functioning

Lastly, we performed a Rasch differential item functioning test to see if respondents conceptualised items differently based on the mode of administration (online versus postal). The reason for this is because there may be pre-existing differences between the two groups that may result in group mean differences and tests like e.g. Kruskal–Wallis would not be appropriate. Rasch differential item functioning test controls for respondent severity before assessing differences in item severity by mode. We defined differential item functioning as a contrast difference > 0.5 and the presence of a significant p-value after adjusting for multiplicity (Bonferroni adjustment).

Scale Reliability/Internal consistency

First, we assessed the internal consistency of the ASCOT-Proxy and ASCOT-Carer using Cronbach’s alpha [18] as it is widely and frequently used reliability index. Next, we also calculated ordinal alpha (introduced by Zumbo et al. [19]), as it was suggested that ordinal reliability coefficients are more suitable for ordinal response scales such as Likert-type response formats (e.g. Cronbach’s alpha underestimates the reliability of the ordinal response scales) [20]. As a minimum, we expected the measures to meet the .70 reliability standard [21].

Construct validity

The present study builds on a previous work of developing the ASCOT-Proxy and ASCOT-Carer. Regarding, the ASCOT-Proxy, previous development phases included: 1. a literature review to identify the methodological challenges and other issues associated with proxy response in the context of self-completion surveys to collect quality of life data for outcomes-based service management, commissioning and policy strategy [22]; 2. focus groups with paid and unpaid (family) carers to explore their views on being a proxy on behalf of the person(s) they care for [23]; and 3. qualitative cognitive interviews with paid and unpaid (family) carers to evaluate the acceptability and content validity of the ASCOT-Proxy [24]. This is the first study reporting psychometric analysis for the ASCOT-Proxy. Compared to the ASCOT-Proxy, the psychometric properties of the ASCOT-Carer have been previously tested among carers in England [25]. However, the study in England only included a relatively small number of carers of people with dementia, so separate subgroup analysis was not possible [25].

In this study, we explored two types of construct validity of the ASCOT-Proxy and ASCOT-Carer: 1. convergent validity, i.e. how similar the measures are to other conceptually-related measures and 2. known-groups validity, i.e. whether the questionnaire can discriminate between groups known to differ on the variable of interest. We assessed construct validity of the questionnaires using hypothesis testing, of which 75 % of hypotheses needed to be correct [26]. Table 1 provides an overview of the variables used for testing of construct validity including the expected strength and direction of the associations. To establish the construct validity of the ASCOT-Proxy, we based all hypotheses on recent validation studies of the ASCOT-SCT4 measure, including adults with long-term physical, sensory and mental health conditions in England [27]; and Austrian home care service users [28]. To establish the construct validity of the ASCOT-Carer, we based all hypotheses on validation studies of the ASCOT-Carer among unpaid carers living in England [25, 29]; among informal carers in Australia [30]; and among informal carers of older adults in Austria [31]. As none of the previous validation studies used DEMQOL measures, we hypothesised the association between the ASCOT-Proxy and ASCOT-Carer and DEMQOL measures based on the association reported between DEMQOL measures and other measures of wellbeing and health.

****Please insert Table 1 below here*******

We used the Spearman correlation coefficient to assess associations between the ASCOT-Proxy and ASCOT-Carer overall scores (continuous variables) and related constructs (continuous variables). We used the correlation coefficient as a measure of the size of the effect. We interpreted values of ± 0.1 as a small effect, ± 0.3 as a medium effect and ± 0.5 as a large effect [32]. We explored known-groups validity using the Kruskal–Wallis test comparing the ASCOT-Proxy and ASCOT-Carer overall scores between subgroups of the sample. Given these are exploratory analyses we did not utilise multiplicity adjustments and applied a two sided p-value <0.05.

Sample size

Data analysed in this paper were collected as part of the ‘Measuring Outcomes of People with Dementia and their carers’ (MOPED) study. As we did not have pilot data for the ASCOT-Proxy with which to calculate a sample size estimate, before we started data collection, we applied a minimum sample size of n=300 needed for the exploratory factor analysis, a recommendation often mentioned by textbooks (e.g. [32]). However, as highlighted by recent publication by Gugiu et al. [5] this is only one of the options when deciding adequate sample size for exploratory factor analysis. In their study [5] they recommended to follow guidance from Monte Carlo simulations examining the impact of ordinal data on exploratory factor analysis [33]. The results from these simulations suggest that when primary factor loadings were medium and the number of factors was 2, a sample size of 200 was adequate [33]. It is important to highlight that this study was originally planned to follow Classical Test Theory methods, which informed sample size calculation. However, the minimum sample size of n=300 is satisfactory for conducting and interpreting Rasch analysis [34].

**Table 1.** An overview of the variables used for testing of construct validity of the ASCOT-Proxy-person and ASCOT-Carer

|  | **ASCOT-Proxy-person*** | **ASCOT-Carer** |
| --- | --- | --- |
| **Variables** | **Anticipated associations** | **Anticipated associations** |
| **Well-Being & Health** |  |  |
| EQ-5D questionnaires [35] of health-related quality of life |  |  |
| EQ-5D-5L (carer) | n/a | Medium [29, 31] to large [30] positive correlation of the EQ-5D index with overall ASCOT-Carer score. |
| EQ-5D-5L-Proxy (proxy-proxy and proxy-person) | Medium positive correlation of the EQ-5D index with overall ASCOT-Proxy-person score [27, 28] | n/a |
| DEMQOL questionnaires |  |  |
| C-DEMQOL [36]: quality of life of carers of people with dementia | n/a | We expect a large positive correlation. This is based on a relationship between C-DEMQOL and other measures of carer’s health and wellbeing [37]. |
| DEMQOL-Proxy [38, 39]: proxy-proxy version of quality of life of people with dementia | We expect a large positive correlation. This is based on a relationship between DEMQOL-Proxy and EQ-5D proxy rating [40]. | n/a |
| Overall quality of life: one item |  |  |
| Carers quality of life | n/a | Medium [31] to large [29] positive correlation between the overall ASCOT-Carer score and overall quality of life. |
| Proxy-proxy and proxy-person | Medium [28] to large [27] positive correlation between the overall ASCOT-Proxy-person score and overall quality of life. | n/a |
| The Carer Experience Scale (CES) [41]: carer-related quality of life | n/a | Large [28, 30] positive correlation between ASCOT-Carer and carer-related quality of life |
| ASCOT-Proxy-person | n/a | We expect small positive correlation between ASCOT-Proxy-person and ASCOT-Carer. This is based on a relationship between DEMQOL-Proxy and C-DEMQOL sum scores [37]. |
| **Satisfaction with social care services** **[42]** (carer measures) | n/a | Medium positive correlation between the overall ASCOT-Carer score and satisfaction with social care services [29]. |
| **Home design suitability for care recipient’s needs**: proxy-proxy perspective | The better the home is designed to meet person’s needs the higher overall ASCOT-Proxy-person score [27]. | The better the home is designed to meet person’s needs the higher overall ASCOT-Carer score [25]. |
| **Impact of caring on health** (yes/no) | n/a | Lower ASCOT-Carer score for those whose health was impacted by the caring situation [25]. |
| **Caregiving situation** |  |  |
| Carer and person with dementia lives in the same household | n/a | Lower ASCOT-Carer scores for those sharing same household [25, 30]. |
| Relationship to a person living with dementia (spouse/partner; parent; sibling; child; other) | n/a | Different overall ASCOT-carer scores based on relationship to a person, e.g. children having the lowest score [30]. |
| Hours of care per week (0-9; 10-19; 20-34; 35-49; 50 or more) | n/a | Different overall ASCOT-carer scores based on hours of spend on caring, e.g. the lowest score for those spending the most hours on caring [25]. |
| Help with personal care (yes; no) | n/a | Lower ASCOT-Carer score for those who help with personal care [25]. |
| Help with giving medicines (yes; no) | n/a | Lower ASCOT-Carer score for those who help with giving medicines [25]. |
| **Sociodemographic characteristics:** |  |  |
| Carer’s gender | n/a | Lower ASCOT-Carer score for female carers when compared to male carers [25]. |
| Carer in paid employment (no; yes including full- and part-time) | n/a | Lower ASCOT-Carer score for those not in paid employment [25]. |
| Instrumental activities of daily living (I/ADLS) (proxy-proxy version) [43]^a^ | Small negative correlation between the (I)ADLS and the overall ASCOT-Proxy-person score [28]. | n/a |

^a^Instrumental activities of daily living (I/ADLS): total number of eight ADLs with difficulty or unable to complete alone (higher the score, the more ADLs with difficulty: getting around (except steps) indoors; getting in and out of bed; eating; paperwork or finances; having a bath or shower; dressing or undressing; using the toilet; washing hands and face)

n/a: not used for validation

*Construct validity was tested only for the ASCOT-Proxy-person as there was an issue with structural characteristics of the ASCOT-Proxy-proxy (two factors solution as opposed to one factor solution). This needs further investigation in future studies.

**An example of steps that we followed in WINSTEPS for the ASCOT-Carer**

Control file name? (e.g., exam1.txt). Press Enter for Dialog Box:

G:\NIHR RfPB Carers of PlWD Data\WINSTEPS\ASCOTCarer.txt

Report output file name (or press Enter for temporary file, Ctrl+O for Dialog Box):

/*Enter*/

Extra specifications (if any). Press Enter to analyse:

ISGROUPS=0 /*To apply partial credit model*/

Processing Table 10 ^^[[1]](#footnote-1)^^

Loading graphing module ....^^[[2]](#footnote-2)^^

Collecting empirical data …

Processing Table 1 ^^[[3]](#footnote-3)^^

Processing Table 3.2+ ^^[[4]](#footnote-4)^^

Processing Table 30 ^^[[5]](#footnote-5)^^

Processing: DIF=@survey /*by survey administration mode: postal or online*/

Processing: DIF=@OP /*by age: under 65 years old versus 65 years or over */

**References**

1. Netten, A., et al., *Outcomes of social care for adults: developing a preference-weighted measure.* Health Technol Assess, 2012. **16**(16): p. 1-166.

2. *EQ-5D*. 25/09/2019]; Available from: <https://euroqol.org/>.

3. *ICECAP-O*. [cited 2023 10/02/2023]; Available from: <https://www.birmingham.ac.uk/research/activity/mds/projects/haps/he/icecap/icecap-o/index.aspx>.

4. Rand Stacey, T.A.-M., Malley Juliette, Silarova Barbora *Exploring the structural characteristics of the Adult Social Care Outcomes Toolkit (ASCOT) and ASCOT-Carer.* NIHR Open Research, 2022.

5. Gugiu, P.C., D. Drew, and E. Polek, *A Critical Appraisal of the Evidence Supporting the Factor Structure of Extant Coping Instruments.* Evaluation & the Health Professions, 2022. **45**(3): p. 235-248.

6. Dinno, A. *paran. Horn's Test of Principal Components/Factors (Parallel Analysis)*. 2015 16/12/2015; 1.5.3:[Available from: <https://www.alexisdinno.com/stata/paran.html>.

7. Gugiu, P.C., et al., *Development and evaluation of the short version of the Patient Assessment of Chronic Illness Care instrument.* Chronic Illness, 2009. **5**(4): p. 268-276.

8. Tennant, A., S.P. McKenna, and P. Hagell, *Application of Rasch analysis in the development and application of quality of life instruments.* Value Health, 2004. **7 Suppl 1**: p. S22-6.

9. Rasch, G., *Studies in mathematical psychology: I. Probabilistic models for some intelligence and attainment tests.* 1960: Nielsen & Lydiche.

10. Shi, Y., et al., *A Rasch Analysis Validation of the Maslach Burnout Inventory-Student Survey with Preclinical Medical Students.* Teach Learn Med, 2019. **31**(2): p. 154-169.

11. Mokkink, L.B., et al., *The COSMIN checklist for assessing the methodological quality of studies on measurement properties of health status measurement instruments: an international Delphi study.* Qual Life Res, 2010. **19**(4): p. 539-49.

12. Tennant, A. and P.G. Conaghan, *The Rasch measurement model in rheumatology: what is it and why use it? When should it be applied, and what should one look for in a Rasch paper?* Arthritis Rheum, 2007. **57**(8): p. 1358-62.

13. Masters, G.N., *A rasch model for partial credit scoring.* Psychometrika, 1982. **47**(2): p. 149-174.

14. Jüttner, M., et al., *Development and use of a test instrument to measure biology teachers’ content knowledge (CK) and pedagogical content knowledge (PCK).* Educational Assessment, Evaluation and Accountability, 2013. **25**(1): p. 45-67.

15. Linacre, J.M., *Optimizing rating scale category effectiveness.* J Appl Meas, 2002. **3**(1): p. 85-106.

16. Pesudovs, K., et al., *The development, assessment, and selection of questionnaires.* Optom Vis Sci, 2007. **84**(8): p. 663-74.

17. McAlinden, C., et al., *Psychometric properties of the NEI-RQL-42 questionnaire in keratoconus.* Invest Ophthalmol Vis Sci, 2012. **53**(11): p. 7370-4.

18. Cronbach, L.J., *Coefficient alpha and the internal structure of tests.* Psychometrika, 1951. **16**(3): p. 297-334.

19. Zumbo, B.D.G., Anne M.; Zeisser, Cornelia *Ordinal Versions of Coefficients Alpha and Theta for Likert Rating Scales.* Journal of Modern Applied Statistical Methods 2007. **Vol. 6** (Iss. 1 ).

20. Gadermann, A.M., Guhn, Martin, Zumbo, Bruno D. , *Estimating ordinal reliability for Likert-type and ordinal item response data: A conceptual, empirical, and practical guide.* Practical Assessment, Research, and Evaluation, 2012  **Vol. 17**.

21. Nunnally, J.C., *Psychometric theory*. 1978): McGraw-Hill.

22. Rand, S.E., Caiels, J., *Using proxies to assess quality of life: A review of the issues and challenges.* 2015, Personal Social Services Research Unit, University of Kent: Canterbury.

23. Caiels, J., et al., *Exploring the views of being a proxy from the perspective of unpaid carers and paid carers: developing a proxy version of the Adult Social Care Outcomes Toolkit (ASCOT).* BMC Health Services Research, 2019. **19**(1): p. 201.

24. Rand, S., et al., *Developing a proxy version of the Adult social care outcome toolkit (ASCOT).* Health Qual Life Outcomes, 2017. **15**(1): p. 108.

25. Rand, S.E., et al., *Factor structure and construct validity of the Adult Social Care Outcomes Toolkit for Carers (ASCOT-Carer).* Qual Life Res, 2015. **24**(11): p. 2601-14.

26. Prinsen, C.A.C., et al., *COSMIN guideline for systematic reviews of patient-reported outcome measures.* Qual Life Res, 2018. **27**(5): p. 1147-1157.

27. Rand, S., et al., *Validity and test-retest reliability of the self-completion adult social care outcomes toolkit (ASCOT-SCT4) with adults with long-term physical, sensory and mental health conditions in England.* Health and Quality of Life Outcomes, 2017. **15**(1): p. 163.

28. Trukeschitz, B., et al., *Cross-cultural adaptation and construct validity of the German version of the Adult Social Care Outcomes Toolkit for service users (German ASCOT).* Health and Quality of Life Outcomes, 2020. **18**(1): p. 326.

29. Rand, S., et al., *Measuring the outcomes of long-term care for unpaid carers: comparing the ASCOT-Carer, Carer Experience Scale and EQ-5D-3 L.* Health and Quality of Life Outcomes, 2019. **17**(1): p. 184.

30. McCaffrey, N., et al., *Head-to-Head Comparison of the Psychometric Properties of 3 Carer-Related Preference-Based Instruments.* Value Health, 2020. **23**(11): p. 1477-1488.

31. Trukeschitz, B., et al., *Translation, cultural adaptation and construct validity of the German version of the Adult Social Care Outcomes Toolkit for informal Carers (German ASCOT-Carer).* Qual Life Res, 2021. **30**(3): p. 905-920.

32. Field, A., *Discovering statistics using IBM SPSS Statistics*. 5th ed. Vol. . 2018, London: : Sage Publications Ltd. .

33. Jin, R., *Sample size in exploratory factor analysis with ordinal data.* . 2012 University of Florida.

34. Linacre, J.M., *Sample Size and Item Calibration Stability.* Rasch Measurement Transactions, 1994. **7**(4): p. p.328.

35. Foundation., E.R., *EQ-5D-5L User Guide Version 3.0 September 2019*. 2019.

36. *C-DEMQOL - a measure of carer quality of life for carers of someone with dementia.* 30/09/2019]; Available from: <https://www.bsms.ac.uk/research/neuroscience/cds/research/cdemqol.aspx>.

37. Brown, A., et al., *Measuring the quality of life of family carers of people with dementia: development and validation of C-DEMQOL.* Quality of Life Research, 2019. **28**(8): p. 2299-2310.

38. Mulhern, B., et al., *Development of DEMQOL-U and DEMQOL-PROXY-U: generation of preference-based indices from DEMQOL and DEMQOL-PROXY for use in economic evaluation.* Health Technol Assess, 2013. **17**(5): p. v-xv, 1-140.

39. Smith, S.C., et al., *Development of a new measure of health-related quality of life for people with dementia: DEMQOL.* Psychol Med, 2007. **37**(5): p. 737-46.

40. Hendriks, A.A.J., et al., *Reliability and validity of a self-administration version of DEMQOL-Proxy.* International Journal of Geriatric Psychiatry, 2017. **32**(7): p. 734-741.

41. *The Carer Experience Scale (CES)*. 30/09/2019]; Available from: <https://www.birmingham.ac.uk/research/activity/mds/projects/HaPS/HE/ICECAP/CES/index.aspx>.

42. Department of Health and Social Care, *Carers strategy: the second national action plan 2014 to 2016.* 2014: London.

43. Malley, J., Caiels, J., Fox, D., McCarthy, M., Smith, N., Beadle-Brown, J., Netten, A., Towers, A-M., *A report on the developmental studies for the National Adult Social Care User Experience Survey* 2010, Personal Social Services Research Unit.

1. Select Output Tables – 10. ITEM Column: fit Order /*Item statistics*/ [↑](#footnote-ref-1)
2. Select Graphs – Category Probability Curves /*Category response curves*/ [↑](#footnote-ref-2)
3. Select Output Tables – 1. Variable Maps /* Rasch Wright Andrich Threshold*/ [↑](#footnote-ref-3)
4. Select Output Tables – 3.2+ Rating (Partial Credit) scale [↑](#footnote-ref-4)
5. Select Output Tables – 30. ITEM: DIF, between/within /*DIF*/ [↑](#footnote-ref-5)
